# Influence of Geomorphic Disturbance on Phenotypic Species Plasticity and Vegetation Cover in High‐Elevated Belts

**DOI:** 10.1002/ece3.73056

**Published:** 2026-03-12

**Authors:** Sarah Kinzner, Katharina Ramskogler, Sophia Castlunger, Florentin Hofmeister, Erich Tasser

**Affiliations:** ^1^ Eurac Research Institute for Alpine Environment Bolzano/Bozen Italy; ^2^ Austrian Academy of Sciences Institute for Interdisciplinary Mountain Research Innsbruck Austria; ^3^ Department of Ecology, Universität Innsbruck Innsbruck Austria; ^4^ Bavarian Academy of Sciences Insitute for Geodesy and Glaciology Munich Germany

**Keywords:** Central Alps, cryophilic species, functional plant traits, geomorphic disturbance, plasticity

## Abstract

The degradation of permafrost and the higher number of heavy rainfall events increase geomorphic disturbance (GMD), affecting the vegetation establishment in high‐elevation belts. To evaluate the effect of GMDs on vegetation development, it is essential to understand how species or plant groups respond to disturbances. In this study, we investigated vegetation establishment in undisturbed and disturbed plot pairs along elevational transects in the Austrian and Italian Central Alps. Differences in total vegetation cover, species diversity, the cover of different plant groups and the community weighted means of the Landolt indicator values were analysed using the Kruskal–Wallis test for non‐parametric data and paired *t*‐test for parametric data. Generalised additive models combined with a principal component analysis were applied to identify the significant environmental variables (e.g., inclination or precipitation) explaining the differences found. To assess species' plasticity, the three most abundant species (five individuals per plot) per undisturbed‐disturbed pairs were collected on‐site, and functional traits measured in the laboratory. Disturbed sites exhibited lower total vegetation cover, species number, cover of competitive species, dwarf shrubs, herbs and lichens. The cover of stress‐tolerant, cryophilic species and herbs was higher in disturbed sites. The observed variations can be mainly explained by climatic, edaphic and topographic variables. The Stream Power Index as a disturbance proxy had a significant negative influence on the total vegetation cover and herb cover and a positive influence on bryophyte, dwarf shrub, and tree cover. Most collected species showed high trait plasticity, with disturbance primarily reducing plant height and specific leaf area. Synthesis: GMD was the key driver in relation to both vegetation cover and species richness. The cover of most functional plant groups as well as species plasticity was primarily affected by climatic factors, soil conditions and the presence of less acidic debris than by GMD.

## Graphical abstract

Climate change increases geomorphological disturbances, which in turn affect vegetation establishment in high‐elevation areas; understanding species' responses to such disturbances is essential. We analysed vegetation across disturbed and undisturbed alpine sites to identify environmental drivers and assess species trait plasticity. While disturbed sites had lower vegetation cover and favoured stress‐tolerant species, vegetation patterns were more strongly shaped by climatic and edaphic conditions than by disturbance intensity.

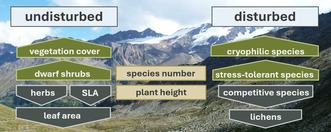

## 1 Introduction

The high mountains are a harsh environment and represent a highly diverse and dynamic landscape. The great topographical and edaphic diversity creates conditions that promote biodiversity and generate unique aesthetic landscapes and vital ecosystem services like clean water, food and climate regulation for surrounding lowlands (Körner et al. 2005; White 1979). In addition, this area is experiencing highly dynamic processes, such as glacier retreat induced by climate change, which is occurring at twice the global average rate (Rumpf et al. 2022). Climate models predict a further increase in temperature, precipitation intensity and frequency of extreme weather events like heatwaves and droughts for this century (IPCC 2023). These predicted changes will have an even greater impact on the melting of snow and ice and the thawing of permafrost (Haeberli and Beniston 1998). As a result, slope stability is becoming increasingly weakened (Brighenti et al. 2019; Reinhardt et al. 2010), resulting in an increase in geomorphic disturbances (GMD), including mass movements such as debris flows, surface erosion and rockfalls (Chiarle et al. 2021; Krainer et al. 2012; Hirschmugl 2003), and in an increase of flood risks in the valleys (Körner 2021; Papthoma‐Köhle et al. 2012). Although numerous studies have documented the effects of warming on biodiversity as a whole and on important taxonomic groups in particular, especially invertebrates, vertebrates and vascular plants (Lenoir et al. 2008; Pauli et al. 2012), the general validity of such responses, taking into account the associated changes in site conditions and GMD, is still unclear. Very few studies have examined how warming affects, for example, the stability of the system and the associated consequences across multi‐taxonomic groups, life forms and strategy types (e.g., Becker‐Scarpitta et al. 2022; Lenoir et al. 2020).

Furthermore, GMD are strongly modulated by the parent rock and the associated pedogenesis and edaphic conditions (Earle 2019; Anderson 1988). Acidic conditions lead to chemical weathering, which breaks down minerals and produces weaker, fine‐grained clay particles that generally promote soil development but also increase pore pressure and reduce soil strength. Alkaline conditions can also lead to instability by causing swelling clays to absorb water, further reducing strength and stability.

GMD not only represents a serious risk to local communities and infrastructure, but it is also a key factor for ecosystems and organism assemblage (Haselberger et al. 2023; Rice et al. 2012; Virtanen et al. 2010). According to the intermediate disturbance hypothesis (IDH; Salminen et al. 2025; Grime 1977), low levels of disturbance promote diversity. IDH's proposal is that the relationship between species diversity and disturbance is hump‐shaped, meaning that intermediate levels of disturbance are optimal for maintaining the highest biological diversity in plant and animal communities (Salminen et al. 2025). At low disturbance levels, strong competitors monopolise resources and outcompete weaker species, while at high disturbance levels, all but the most resilient species are eliminated. In extreme cases, however, disturbance can cause the total loss of established vegetation. This can result in the creation of so‐called ‘vegetation gaps’ (Nash Suding and Goldberg 2001). These gaps lead to changes in abiotic soil conditions and competition dynamics (Nash Suding and Goldberg 2001), which in turn cause changes in species and functional diversity, as well as in community composition (Haselberger et al. 2024; Eichel et al. 2023; Kemppinen et al. 2022; Smith et al. 2022). However, the specific factors determining which species establish under disturbed versus undisturbed conditions remain largely unresolved.

Evolutionary selection pressures have driven morphological and physiological adaptations in plants, which can be studied at molecular and genetic levels (Price et al. 2003; Zhang et al. 2022). Examining the resulting plant traits provides valuable insights into how plant species react to slow changes in climate and edaphic conditions with regard to resource strategies and competitiveness (Smith et al. 2022). In general, at such high‐elevated sites, cryophilic species, which are adapted to low temperatures and very low levels of limiting resources, are the predominant species (Kobiv 2018; Stoica et al. 2018). At the same time, however, the highly dynamic nature of mountain environments also requires flexible and rapid acclimatisation to current environmental conditions (Halbritter et al. 2018; Keiler and Fuchs 2016; Gentili et al. 2013; Hoffmann and Sgrò 2011), which is made possible by phenotypic plasticity (Sommer 2020). The extent to which both adaptations and acclimatisation contribute to survival in high mountains has hardly been researched.

This study aimed to investigate how GMD affects subalpine–nival vegetation and drives species acclimatisation through trait variation. Thus, we hypothesised:

- Environmental conditions differ significantly between disturbed and undisturbed areas, leading to changes in vegetation cover and species richness.
- GMD significantly shapes patterns of functional plant groups (life forms, strategy types and temperature types) and trait variability.
- The high degree of phenotypic plasticity evident in alpine plant species results in shorter growth, smaller leaves and a reduction in specific leaf area (SLA) in disturbed areas.

To test our hypotheses, we compared disturbed and undisturbed sites in terms of vegetation cover, species diversity, and the relative cover of strategy‐types, functional plant groups, and thermophilic and cryophilic species, as well as morphological traits of highly abundant species. The relative cover of the different functional groups was calculated as the sum of the cover of the single species (functional groups) divided by the sum of the cover of all species multiplied by 100, whereby only cover is used as a synonym in the following text.

## 2 Materials and Methods

### 2.1 Study Area

**FIGURE 1:**
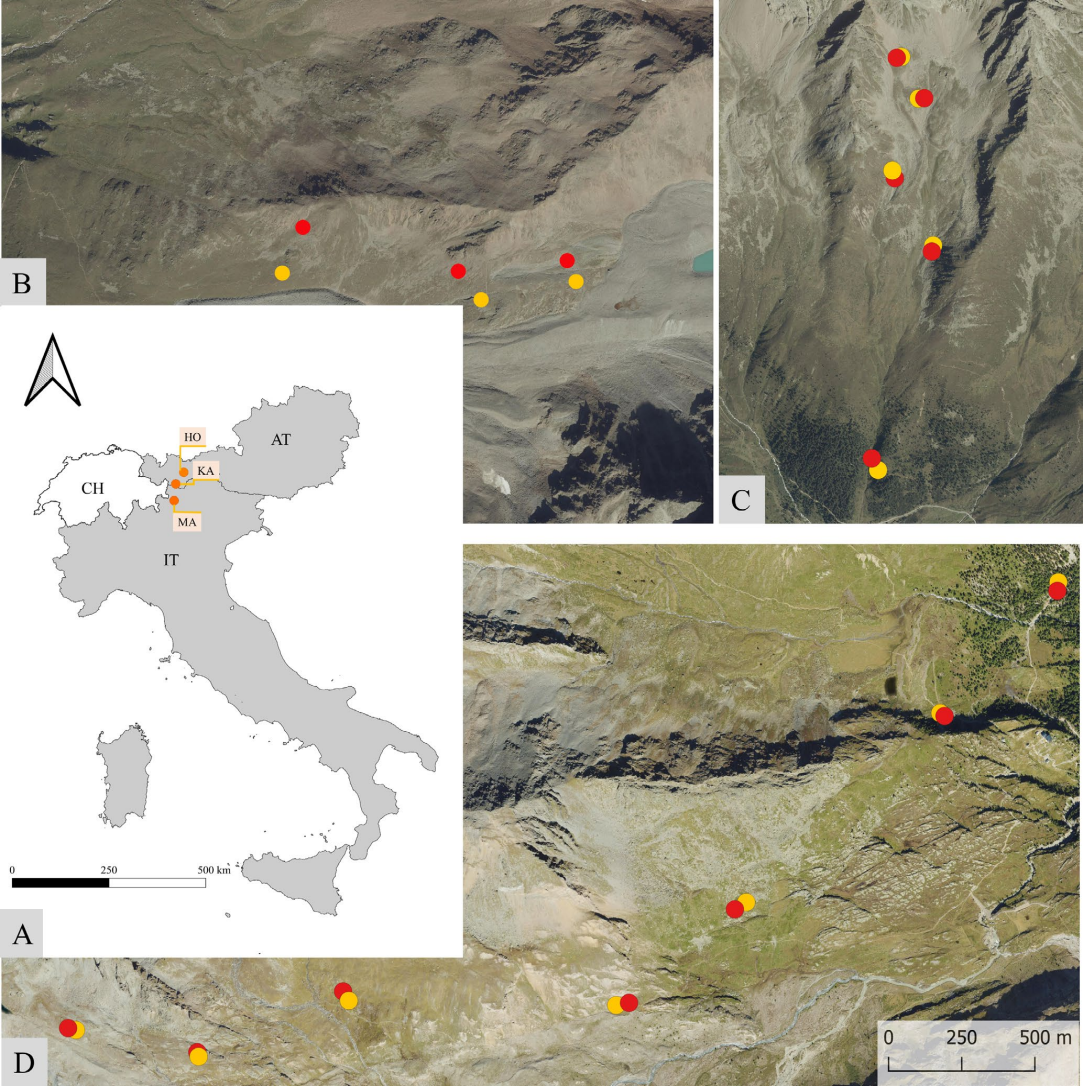
(A) Study areas in Italy (IT) and Austria (AT) (Runfola et al. 2020) and the elevation transects with undisturbed (yellow) and disturbed (red) plots in the Kauner Valley (KA, B; source of orthophoto: Government of Tyrol, Austria), Horlach Valley (HO, C; source of orthophoto: Government of Tyrol, Austria 2024), and Martell Valley (MA, D; source of orthophoto: Autonomous Province of Bozen/Bolzano, Italy 2023).

We conducted fieldwork in three valleys in the Central European Alps (Figure 1A): Kauner Valley (Figure 1B), Horlach Valley (Figure 1C; both Tyrol/Austria) and Martell Valley (Figure 1D; South Tyrol/Italy). The Kauner Valley is located in the Ötztaler Alps and oriented in a north–south direction. The area is geologically characterised by the Eastern Alps crystalline complex, also known as the Ötztal‐Stubai complex (Tollmann 1977). It mainly consists of acidic para‐ and ortho‐gneisses, amphibolites, and mica schists (Tollmann 1977; Vehling 2016). The Horlach Valley forms an east–west oriented side valley of the Ötz Valley in the Stubai Alps and is also geologically influenced by the acidic Ötztal‐Stubai Complex (Bögel and Schmidt 1976; Tollmann 1977). The third study area, the Martell Valley, is a southwest‐northeast oriented side valley of the Vinschgau, located in the Ortler‐Cevedale group. The area is dominated by the acidic Ortler‐Campo crystalline, which is mainly composed of metamorphic rocks like mica schists, amphibolites and orthogneisses, with an influence of the alkaline marble (Mair et al. 2007; Nocker et al. 2004). Due to their location in the Central Alps, all the study areas are characterised by a continental, dry inner‐Alpine climate (‘3pclim’ 2024; Galos 2017; Heckmann et al. 2016). An overview of the three study areas is given in Tables 1 and S1.

**TABLE 1:** Characterisation of the transects in the study areas: Location (coordinates of the centre of the transect, EPSG: 25832), geology (Mair et al. 2007; Nocker et al. 2004; Tollmann 1977; Bögel and Schmidt 1976), elevation, aspect, inclination (the one for Kauner Valley and Horlach Valley derived from the DTM 2017 and the one for Martell Valley from the DTM 2019, all provided by the Physical Geography, Catholic University [KU] Eichstaett‐Ingolstadt), mean annual temperature and mean annual sum of precipitation (both for the period 1981–2010, 3pclim) are given.

	Kauner Valley	Horlach Valley	Martell Valley
Location	633889.3/5195099.4	652785.1/5225631.0	627552.3/5148115.3
Geology, parent material	Acidic	Acidic	Acidic/alkaline
Elevation (m a.s.l.)	2441–2801	2118–2737	2207–2857
Aspect	W‐SW	SW‐S	NE, SE, S
Inclination (°)	20.4–35.5	13.1–37.8	9.2–57.1
Temperature (°C)	−1.1–0.6	−1.9–1.7	−1.0–2.4
Precipitation (mm)	1118–1154	951–1011	1148–1307

### 2.2 Study Design

#### 2.2.1 Vegetation Surveys

We installed 30 sample plots along the three elevation transects: 15 undisturbed and 15 disturbed (see Figure 1, Table S2). As the plots in Kauner Valley (*n* = 6; 20/2022) and Martell Valley (*n* = 14; 2019/2023) were surveyed twice, and the samples in Horlach Valley (*n* = 10; 2022) were surveyed only once, the total sample size is 50 surveys, of which 25 were conducted in undisturbed areas and 25 in disturbed areas. The 10 × 10 m plots were distributed at regular intervals of approximately 100 m of elevation along the transects, with the disturbed and undisturbed plots selected near each other (< 70 m) and at comparable topographic characteristics (e.g., inclination, aspect and elevation). We selected the pairs of plots along elevation gradients to be able to address phenotypic plasticity due to GMD in different altitudinal belts and also different vegetation types (plant communities). Disturbed areas refer to areas affected by GMDs, primarily gravitational, nival, periglacial, weathering and soil erosion processes. The differentiation was made on‐site based on visible evidence such as rockfall, snow scraping and avalanche damage to the vegetation cover, as well as visible solifluction and topsoil erosion. Furthermore, we identified them also by utilising the different landforms and surface textures. When selecting the plots on site, however, care was taken to ensure that they were not new, fresh disturbance areas, but that the disturbance was permanent and long‐term in nature. The partial colonisation of the disturbed areas was used as a criterion for this. The general rule for selection of the exact location was that, for plots with the same site characteristics, the vegetation cover between disturbed and undisturbed plots must (a) differ by at least 15% and (b) the disturbed area must have a minimum total unvegetated area of 30% (ideally between 60% and 98%).

For each plot, total vegetation cover, as well as the cover of all vascular species (Table S2), bryophytes, and lichens was estimated in the field in percentage. The cover of the single species was recorded using the extended scale according to Braun‐Blanquet (Reichelt and Wilmanns 1973). These values were then converted into mean cover values (%) (Van der Maarel 1979). The plant species were identified according to Fischer et al. (2008) and standardised based on the nomenclature according to the World Flora Online (World Flora Online, www.worldfloraonline.org, accessed between September and December 2024).

The cover of thermophilic and cryophilic species, as well as of the different lifeforms (i.e., bryophytes, lichens, graminoids, herbs, dwarf shrubs and trees), was calculated as the sum of the cover of the individual species (functional groups) divided by the sum of the cover of all species multiplied by 100. The cover of trees also includes shrubs and is within the text referred to as the cover of trees. Cryophilic species are species with a Landolt indicator value (Landolt et al. 2010) for temperature of 1 and 1.5, thermophilic species with > 1.5 following Evangelista et al. (2016). Additionally, the species' membership of the CSR (competitor, stress‐tolerator, ruderal) strategy types (Grime 1977) was extracted from Flora indicativa (Landolt et al. 2010) and their cover calculated. Competitive species grow best in relatively stable and productive habitats, whereas stress‐tolerant species are adapted to shifting conditions and environments with limited resources. Ruderal species have adjusted to disturbances by investing mainly in their reproductive organs. The combination of the three letters ‘ccc’ stands for a competitive species, ‘rrr’ for a ruderal species, and ‘sss’ for a stress‐tolerant species. Combinations of two of the letters, such as ‘ccr’ or ‘crr’, represent intermediate strategies between the two types. ‘crs’ represents an intermediate strategy between all three types. Afterwards, the species were recognised into four groups (i.e., competitive species [ccc, ccr, ccs], stress‐tolerant species [sss, rss, css], ruderal species [rrr, crr, rrs] and indifferent species [csr]) and their cover calculated.

#### 2.2.2 Site Characteristics

Topographic explanatory variables for all plots were calculated based on digital terrain models with a resolution of 10 m (i.e., the one from Kauner Valley—DTM 2017, Physical Geography, Catholic University (KU) Eichstaett‐Ingolstadt, Horlach Valley—DTM 2017, Physical Geography KU Eichstaett‐Ingolstadt and Martell Valley—DTM 2019, Physical Geography KU Eichstaett‐Ingolstadt), including elevation, inclination and aspect (transformed into northness and eastness using trigonometric functions after Dial (2017)). The topographical information for each plot is reported in Table S2. Additionally, we calculated the SPI, a hydro‐geomorphic parameter as a proxy for disturbance (Table S2).

As meteorological data, we used the 5‐year mean of the annual sum of precipitation and the mean annual temperature calculated from daily data. These data are based on meteorological observations from several stations. The metadata of the respective meteorological stations are listed in the Tables S3–S5. We used the fully distributed Water Flow and Balance Simulation Model (WaSiM) version 10.04.07 (Schulla 2021) for inter‐ and extra‐polating daily and annual temperature and precipitation to a 25 × 25 m grid resolution (for more details see Hofmeister et al. 2022).

Furthermore, we calculated the community weighted means (CWM) of the different Landolt indicator values (Landolt et al. 2010), based on the cover of the plant species, to characterise the site conditions (Table S6). Specifically, we calculated light availability (L), temperature (T), and soil conditions like humus (H) and nutrient content (N), soil reactivity (R), soil dispersion (D), and soil moisture (F). Among other studies, the suitability of indicator values as proxies for soil parameters was described by, for example, Descombes et al. (2020), Simon et al. (2020) and Anschlag et al. (2017).

#### 2.2.3 Morphological Plant Traits

To measure the intraspecific trait variability, plant individuals were collected from the Kauner Valley in 2020 and 2022, from the Horlach Valley in the year 2022, and from the Martell Valley in 2019 and 2023. Five representative individuals of the three most abundant species (mean cover per transect pair 6.36%), occurring in both the disturbed and undisturbed plots per transect pair along the elevation transect, were collected in the field. The selection of species was therefore site‐specific, which meant that the species assemblage could differ between sites and plot pairs (see Table S7). The plant species included competitive, stress‐tolerant and ruderal strategists, as well as different functional groups in response to different environmental conditions. The varying degrees of morphological characteristics are the result of the expression of certain genes, although the underlying genes remain constant (Kumar et al. 2020). Per plot, a total of at least 15 trait replicates and a maximum of 27 trait replicates were collected across the three species. This results in a total of 155 trait replicates for the Kauner Valley, 239 for the Horlach Valley, and 350 for the Martell Valley, that is, a total of 744 replicates across all project areas (of which 370 are for disturbed and 374 for undisturbed sites). The differences between the valleys are due to the number of plots sampled. We wrapped the samples on site in wet paper towels and kept them sealed in plastic bags. They were stored in the freezer until laboratory measurements. The following traits were measured according to Pérez‐Harguindeguy et al. (2013):

- *Stretched plant height* (cm): for rosette plants, the maximum height of the rosette leaves, and for herbaceous plants, the ‘stretched length’ from the root base to the tip of the youngest fully expanded leaf.
- *Leaf area* (mm^2^): three to five representative leaves from each individual were scanned with a millimetre paper below each leaf, and their area was measured using the image analysis software ‘ImageJ’ version 1.8.0 (Schneider et al. 2012) and calibration using the millimetre paper as also described in (Bertel et al. 2022; Peguero‐Pina et al. 2016). In the case of very small leaves (< 25 mm^2^), 5–10 leaves were scanned together, and a mean leaf area was calculated.
- *Leaf dry weight* (mg): the scanned leaves were dried at 80°C for at least 72 h until a constant weight was achieved and then stored at 60°C until weighing. To determine the weight, either an analytical balance (Sartorius Entris 124‐1S) or, in the case of very low leaf weights, a precision balance (Kern EG 220‐3NM, Min. = 0.001 mg; Kern & Sohn GmbH, Balingen, Germany) was used. In the case of leaves with very low weights, five to 10 leaves were weighed together, and the mean weight was calculated.
- *SLA* (mm^2^mg^−1^): we calculated the ratio between leaf area and dry weight.

Leaf area, leaf dry weight and SLA reflect the plant economics spectrum and are also related to disturbance and functional strategies under geomorphic stress (Haselberger et al. 2024; Zanzottera et al. 2020). In the early stages of succession, acquisitive and fast‐growing traits (high SLA, high leaf area, low leaf dry weight, low plant height) were evident, facilitating rapid progression towards a closed canopy and enhanced sediment retention through increased hydraulic roughness. Towards the end of succession, more conservative and stress‐tolerant traits prevailed (Zanzottera et al. 2020). These usually form a dense, tall canopy, reducing exposure to water flow and sediment transport and thus reducing vulnerability and response to geomorphic processes (Haselberger et al. 2024; Kervroëdan et al. 2018).

### 2.3 Statistical Analyses

Statistical analyses were performed using the R software version 4.3.2 (R Core Team 2023). The boxplots were made with the R package ggplot2 (Wickham 2016). As explanatory variables, we used the described topographic, climate and site conditions. Since many of the explanatory variables were highly correlated, a PCA (Data S1) with varimax rotation and using Kaiser's criterion of one was performed to avoid multi‐collinearity (Table S8a,b). The 10 explanatory variables were reduced by PCA to three rotated components (RC1–RC3) with a total explanation of 81% of the variance. RC1 included, among others, Landolt indicator value T (0.96), elevation (−0.87) and mean annual temperature (0.74). Therefore, RC1 was specified as climate‐induced growth. RC2 included primarily edaphic parameters such as nutrient availability (0.85), continentality (−0.76) and water availability in the soil (0.75). Increasing values of the components thus reflect improved edaphic conditions. RC3 was positively correlated with pH (0.86) and dispersity (0.72) and negatively correlated with humus (−0.67). This component will be referred to as less acidic debris sites. The variables SPI, northness, eastness, inclination and precipitation did not load sufficiently into components RC1–RC3, nor were they correlated with each other. Therefore, they were separately used as further explanatory variables in the GAM. Furthermore, we added the factor GMD with two levels (undisturbed and disturbed) as an explanatory variable.

To assess the influence of various explanatory variables on the dependent variables (species richness, total vegetation cover, cover of cryophilic and thermophilic species, cover of competitive, ruderal, stress‐tolerant, and csr‐strategists, cover of trees, dwarf shrubs, graminoids, herbs, bryophytes and lichens, as well as plant height, dry weight, leaf area and SLA), generalised additive models (GAMs) were performed. To enable fitting the GAM with a beta distribution, we transformed the percentage values of total cover and cover into a range between 0 and 1 (Ferrari and Cribari‐Neto 2004) using the following formula:

$$y^{\mathrm{cov}}=\frac{y\times \left(n-1\right)+0.5}{n}$$

*y* ^cov^ represents the transformed ratio of vegetation cover, *y* the cover percentage divided by 100 and *n* the number of plots (Smithson and Verkuilen 2006).

For analysing the effects of the environmental variables on plant traits, GAMs were set up using species as a random effect, which creates a random intercept for each species and allows for adjustment for species‐level variation. The models and model diagnostics are available in the Equations S1–S18; Figure S1–S18.

To investigate statistical differences in the mean cover of life forms and strategy‐types, species richness as an indicator for alpha‐diversity, and plant traits, paired *t*‐tests were conducted for parametric data, and a Kruskal–Wallis test for non‐parametric data. Normality was tested using the Shapiro–Wilk test, and the Levene test was used for testing the homogeneity of variances. Furthermore, to analyse the relationships between the different species groups and the plant traits, the species‐specific trait values were ‘z‐standardised’ (mean‐centred and scaled by standard deviation) to make them comparable when modelling the effects of disturbance on traits across all species. This process enables us to integrate species that differ in their numerical trait range into a single analysis (see also Kothari and Schweiger 2022; Hermes et al. 2016).

## 3 Results

### 3.1 Differences Between Disturbed and Undisturbed Sites in Vegetation and Site Characteristics

Sites affected by GMD showed significantly greater soil dispersion, higher light availability, lower humus content and higher temperature, as shown by the weighted Landolt indicator values (Landolt et al. 2010; Table 2). In addition, the nutrient content was almost significantly (*p* = 0.08) higher on the disturbed areas. There was no statistically significant difference in terms of moisture, continentality and reactivity.

**TABLE 2:** Differences in the community weighted means (CWM) of the Landolt indicator values (Landolt et al. 2010) between disturbed and undisturbed sites (*n* = 50, undisturbed = 25, disturbed = 25).

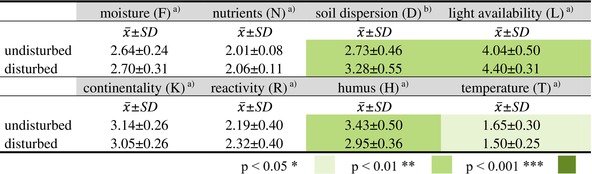

*Note:* Statistically significant differences applying ^a^Kruskal–Wallis tests or ^b^ *t*‐test are given in different colour shades.

Abbreviations: $\bar{x}$ = mean, SD = standard deviation.

The total vegetation cover in disturbed plots was found to be 41.6% significantly lower compared to undisturbed ones (Table 3). The lower total vegetation cover is in line with the higher amount of unvegetated area, which was used as an indicator for GMD. Similarly, the cover of vascular plants was found to be 53.5% lower. Undisturbed sites showed a mean total cover of 82.2% and a mean vascular cover of 81.9%, while disturbed sites only reached 40.6% and 28.5%, respectively. This trend was observed in all three study areas. Species richness was also, with 28.2 species, significantly lower in the disturbed plots (Table 3). In addition, we found a 12.08% higher cover of cryophilic species (e.g., *Agrostis rupestris* or *Helictochloa versicolor*) in disturbed plots (Table 3). However, the cover of thermophilic species (e.g., *Avenella flexuosa* or *Cerastium arvense* ) was 12.08% higher in undisturbed sites. Stress‐tolerant species (e.g., *Saxifraga paniculata* or *Silene rupestris* ) were the most common in both disturbed and undisturbed areas, followed by competitive species (e.g., *Leucanthemopsis alpina* or *Potentilla aurea*; Table 3). While the cover of stress‐tolerant species was 14.09% higher in disturbed areas, the cover of competitive species decreased by 13.61% in disturbed areas (Table 3). Ruderal species (e.g., *Euphrasia minima*) were only weakly represented, with about 3% in both disturbed and undisturbed areas (Table 3). Indifferent species (e.g., *A. rupestris* or *Anthoxanthum odoratum* agg.) showed roughly the same cover (18%) in disturbed and undisturbed areas (Table 3).

**TABLE 3:** Differences in cover between disturbed (1) and undisturbed (0) sites. Statistically significant differences are given in different colour shades, mean ± standard deviation. For temperature types, as well as for the different strategy types and the life forms, relative cover was given.

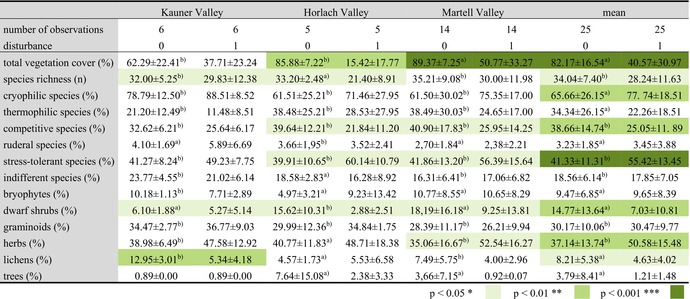

*Note:* Statistically significant differences applying^a^ Kruskal–Wallis tests or ^b^ *t*‐test are given in different colour shades.

In undisturbed areas, herbs covered 37.15%, followed by graminoids with 30.17% cover (Table 3). In disturbed areas, the difference between these two life forms increased: the cover of graminoids was the same, while the cover of herbs increased by 13.44% (Table 3). The cover of dwarf shrubs and lichens was lower in disturbed sites (Table 3).

### 3.2 The Impact of GMD on Functional Plant Groups

Disturbed plots with a high SPI were negatively correlated with total vegetation cover, and the cover of lichens, herbs (non‐linear) and trees (Tables 4, S9a and Figures S19, S20). In addition, there were significant positive correlations between disturbance and the cover of bryophytes (non‐linear) and dwarf shrubs. Climatic, edaphic and topographic variables were important for more different lifeforms and strategy‐types in comparison to the factor GMD and the SPI. Under more favourable climatic growth conditions (RC1), competitive and thermophilic species, dwarf shrubs, graminoids and trees (including shrubs) were promoted as well as total vegetation cover and species richness (Tables 4, S9a and Figure S19). In contrast, the cover of ruderal, stress‐tolerant and cryophilic species, as well as bryophytes, lichens and herbs declined. Favourable edaphic growth conditions (RC2) favoured the cover of herbs (Tables 4, S9d). The cover of competitive species, bryophytes, lichens and trees including shrubs was negatively affected. When the conditions became more alkaline and better aerated (e.g., less acidic debris), the cover of stress‐tolerant and cryophilic species, as well as herbs, increased; the cover of competitive species, dwarf shrubs, and trees and shrubs decreased (Tables 4, S9b,c and Figure S19).

**TABLE 4:** Results of generalised additive models, effects of environmental variables on total cover, species richness, cover of different strategy types, life forms and temperature types, SPI = Stream power index and GMD = geomorphic disturbance, for the parametric coefficients the estimate is given and for the approximate significance of smooth terms the graph only for significant parameters (*R*‐sq. Adj. = *R*‐square adjusted; dev.‐expl. = deviance explained).

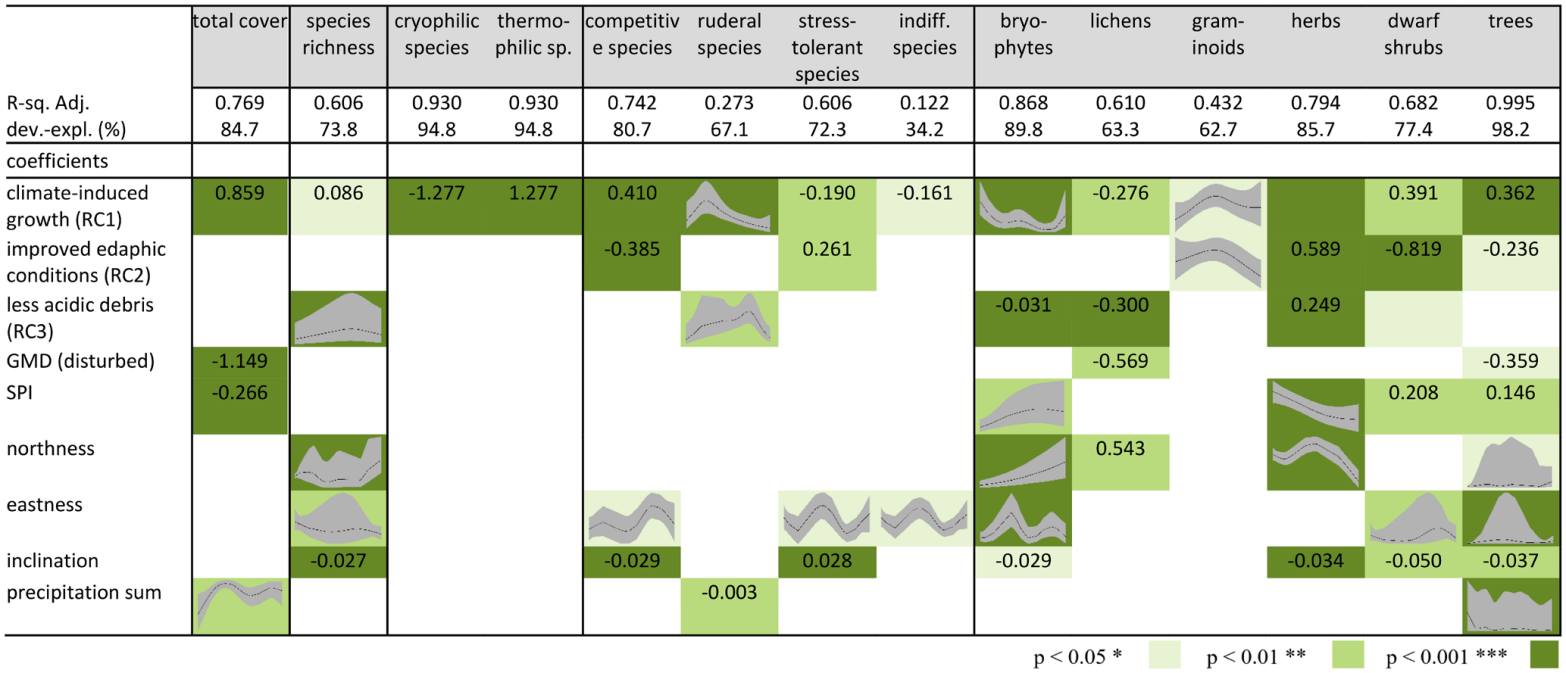

Furthermore, of the topographical variables, east‐facing slopes favoured the growth of dwarf shrubs but had a negative impact on the cover of bryophytes and herbs. Trees and tall shrubs are also increasingly found on eastern and southern slopes. As slope steepness increased (between 35° and 55°), the cover of stress‐tolerant species and herbs rose, while the cover of competitive species, indifferent species and lichens declined. Finally, higher mean rainfall amounts favoured the cover of competitive species and dwarf shrubs, while negatively affecting the cover of ruderal species and trees.

Table S9a–d in the supplement lists the results of the generalised additive models and the approximate significance of the smooth terms. The trends of the smoothed terms are given in Table 4.

### 3.3 The Impact of Environmental Variables and Disturbance on Functional and Species‐Specific Plasticity

By investigating the effects of environmental variables on the different plant traits, we observed a significant positive correlation between RC1 and dry weight, leaf area, and SLA. RC2 showed a positive correlation with dry weight and a negative correlation with SLA, while RC3 showed a positive correlation with leaf area and SLA and a negative correlation with plant height (Tables 5, S10). The GMD and the SPI as proxy for disturbance showed a negative correlation with plant height and leaf area (Tables 5, S10) as well as a non‐linear significant effect on leaf dry weight and a linear one on SLA (Tables 5, S10, Figure S21). Furthermore, some significant correlations between northness and eastness and the different plant traits were detected. The inclination and precipitation were correlated with all traits, whereby the correlation was always non‐linear (Tables 5, S10, Figure S21). Plant height, dry weight and SLA varied across different life forms. Finally, species had a significant effect on all traits. For all traits, the random effect estimates for species are given in the Figure S22 and Table S11 indicates the species.

**TABLE 5:** Results of generalised additive models, effects of environmental variables plant height, leaf dry weight, leaf area and SLA (specific leaf area), SPI = Stream power index and GMD = geomorphic disturbance, for the parametric coefficients the estimate is given and for the approximate significance of smooth terms the graph only for significant parameters (*R*‐sq. Adj. = *R*‐square adjusted; dev.‐expl. = deviance explained).

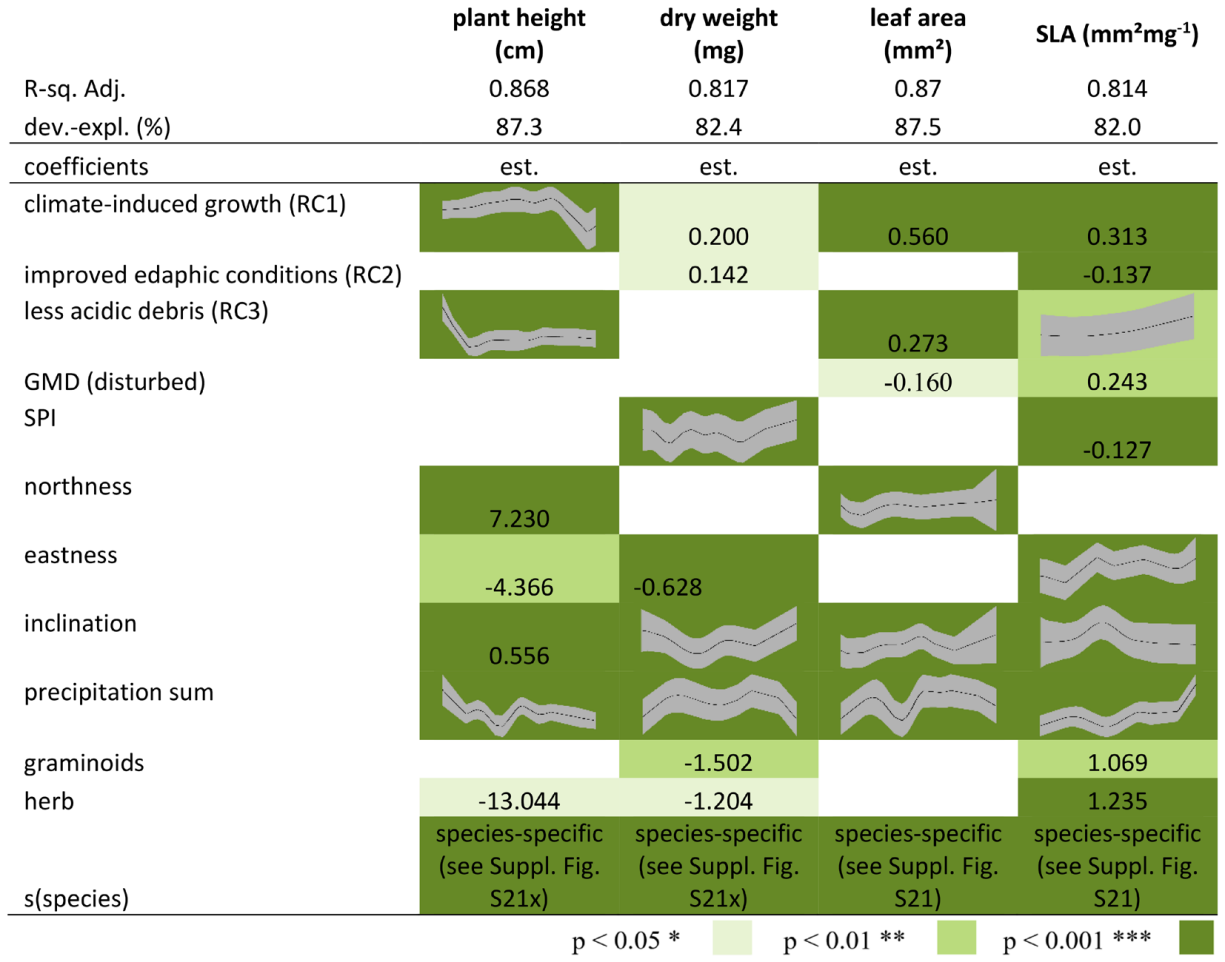

Cryophilic species showed no significant difference in morphological plant traits between undisturbed and disturbed sites (Table 6). In contrast, thermophilic species grew on average 7 cm higher in undisturbed sites (*p* < 0.01), while the dry weight was higher and the leaf area was smaller. The collected competitive species showed a higher growth in undisturbed sites and a higher SLA but were not affected in the other plant traits. Stress‐tolerant species formed lighter, larger leaves in disturbed sites. In contrast, indifferent species in disturbed locations tended to have larger, heavier leaves and a significantly lower SLA. Across all species, there was a reduction in plant height and leaf area, and a tendency towards lower leaf mass and SLA in the disturbed areas (Table 6).

**TABLE 6:** Differences in the plant traits of the different temperature types and strategy types; GMD = geomorphic disturbance with 0 = undisturbed and 1 = disturbed. Statistically significant differences applying Kruskal‐Wallis‐tests are given in different colour shades.

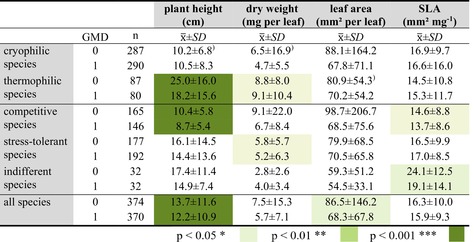

At the species level, we observed intraspecific differences in *A. odoratum* agg., *Thymus praecox* ssp. *polytrichus* and *Juniperus communis var. saxatilis*. They showed differences in all four traits (Table S11). In contrast, the grasses *Oreochloa disticha* and *H. versicolor* and the herb *Phyteuma hemisphaericum* showed no significant differences in any of the traits. Grass species that exhibited significant differences between undisturbed and disturbed sites were mostly taller on the undisturbed ones, with *Juncus trifidus* being the only exception (Table S11). Taller dicotyledons (> 6 cm: *Cardamine resedifolia*, *Geum montanum*, *S. paniculata* , *T. praecox* ssp. *polytrichus*, *Jacobaea incana*, *S. rupestris* ) were found to grow taller and produce larger, heavier leaves with a higher SLA in undisturbed sites. On the other hand, shorter dicotyledons (< 6 cm: *Primula glutinosa*, *Saxifraga bryoides*, *Veronica bellidioides*) performed better on disturbed sites (Table S11).

## 4 Discussion

In this article, we introduce an empirical study analysing the effects of GMD on the development of different life forms, strategy types and temperature types, as well as on the species' reaction to disturbances through short‐term morphological plasticity. For this, plant height, leaf area and SLA were compared among individuals of the same species growing in disturbed and undisturbed areas along elevation gradients in three different study areas in the European Alps.

### 4.1 Changed Site Conditions and Vegetation Cover due to GMD

Our first hypothesis, suggesting significant differences between disturbed and undisturbed sites in terms of growing conditions, vegetation cover, and species and functional diversity, is partially supported by our results. Sites affected by GMD showed greater soil dispersion, higher light availability, lower humus content, higher temperature and lower nutrient content. However, these site variables were not measured in our study but rather derived from the Landolt indicator values (Landolt et al. 2010). Therefore, the resulting findings must be interpreted with some caution. We also found lower total vegetation cover and a higher cover of thermophilic and stress‐tolerant species, as well as lower cover of cryophilic and competitive species on disturbed sites. Nevertheless, due to the design, less total vegetation cover is to be expected in disturbed sites. The shifts observed in the functional groups (more thermophilic and stress‐tolerant species, fewer cryophilic and competitive species) also correspond well with conceptual expectations. In disturbed areas, the graminoid cover was approximately 30%, which was the same as in undisturbed sites. However, the herb cover increased by 13%, accounting for over 50% of the total vegetation cover. The cover of dwarf shrubs and lichens, on the other hand, was lower in disturbed sites.

Previous studies also concluded that GMD, such as erosion, deposition and mixing, alter the soil's chemical, physical and biological properties (Wojcik et al. 2021). In general, higher soil moisture and temperature, as well as the availability of phosphorus (P) and nitrogen (N), promote the speed of pedogenesis, particularly in the accumulation/degradation of soil organic matter and mineral weathering. This, in turn, naturally supports the rate of vegetation development in proglacial areas (Burga et al. 2010; Zimmer et al. 2024). Subsequently, the production and decomposition rates of plant biomass are key drivers to the further build‐up of soil organic matter. If this development is affected by GMD, it is not surprising that this leads to reduced vegetation cover (Eichel et al. 2013; Giaccone et al. 2019). Due to the reduced vegetation cover, the shading of small plant species (e.g., bryophytes) by taller species in disturbed sites is reduced (see Bohner et al. 2019). This species shift to more shade‐intolerant species also affects the light indicator value, which in our case increases significantly as a result of the disturbance. Ellenberg's indicator value is therefore cited in various studies as a good predictor of light conditions in the canopy (e.g., Szymura et al. 2014). The amount of cover depends on the intensity and also frequency of the GMD, and thus on how many plant parts or entire individuals are removed or die. Only when the system has stabilised can the open soil be colonised or re‐colonised through succession, and the vegetation cover increases (Giaccone et al. 2019; Jiao et al. 2009). Our disturbed sites also had slightly fewer species (28.2 species on average) than the stable sites (34.1 species). This is consistent with other studies, which also showed a consistently higher species number with increasing vegetation cover on undisturbed sites, with a peak in species numbers between 40 and 80 years in the proglacial areas (Eichel et al. 2013; Li et al. 2021). In contrast to our study, these studies were often performed in proglacial areas. Proglacial areas are systems transitioning from glacial to non‐glacial conditions. They are in the early stages of soil development and vegetation succession and are characterised by high rates of morphodynamic sediment reworking (Ohler et al. 2023; Haselberger et al. 2021). Our sample sites are all located outside the proglacial zone. Soil and vegetation development are usually well developed, which means that biomass production, for example, is greatly increased. This leads to more humus formation, but also to an increase in soil microbial activity (Thakur et al. 2021). The introduction of organic matter and root exudates into the soil enhances soil aggregation processes, which are important for soil stabilisation (Ohler et al. 2023). Disturbances in such areas are therefore usually small‐scale, and the eroded areas differ from the proglacial areas by frequently having a dense surrounding vegetation cover and a more abundant seed bank (Erschbamer et al. 2001). It is therefore not surprising that a large number of species from the adjacent areas can be found in our disturbed areas, and that the species richness is moderate, but statistically significant, lower.

### 4.2 Effects of GMD on the Cover of Plant Community Composition

The second hypothesis assumed that GMDs are a key factor in the establishment of certain life forms, strategy types and temperature types. Our results showed that disturbance in the form of the indicator GMD and SPI had an effect on the total vegetation cover, the cover of Bryophytes, lichens, herbs, dwarf shrubs and trees, but not for graminoids. However, caution should also be used when interpreting SPI results. The SPI does not directly quantify erosion intensity, but only the susceptibility of a site based on slope and drainage area. The actual thresholds for instability and thus also for the SPI as an erosion indicator vary considerably depending on soil and vegetation.

Disturbance intensity, therefore plays a pivotal role low disturbance intensity does not destroy vegetation but alters species composition (Rehberger 2002), while high disturbance intensity or frequency prevents plant colonisation or allows only the establishment of pioneer and less competitive species (Curry et al. 2006; Eichel et al. 2023; Wojcik et al. 2021). It is well known that firstly pioneer and ruderal species, and afterwards stress‐tolerant species, which are particularly resilient and have effective long‐distance dispersal mechanisms, high reproductive potential and productivity, are the first to become established in an early phase (Eichel et al. 2013; Geitner et al. 2021; Wirth et al. 2024). This is also reflected in our results, although a detailed interpretation is not possible due to a lack of information on the history of disturbances (time since the last disturbance, frequency and extent). However, the history in particular is likely to have a strong influence on the current vegetation pattern. Pioneer species play a key role in building and stabilising the substrate through their root systems and through organic matter accumulation (Greinwald et al. 2021). At the same time, the amount of organic material, nutrient content and microbial activity also rises. These soil factors change in a way that favours the growth of less resilient species, resulting in the displacement of the original pioneer communities. The establishment of more advanced successional stages and the appearance of competitive species (C‐strategists) are linked to more favourable and secure site conditions (Chase and Myers 2011; Del Moral 2009; Kershaw and Mallik 2013). Therewith, the influence of climatic and edaphic factors diminishes (Raab et al. 2012). At the same time, a higher degree of cover makes the site less susceptible to surface erosion (Martin et al. 2010). Our findings are also consistent with these changes: competitive species were significantly more common in undisturbed sites than in disturbed ones. The developments in relation to thermophilic and cryophilic plant species should also be seen in the context of competition. In principle, studies show that the abundance of cryophilic species increases with increasing elevation and the abundance of thermophilic species decreases (Gottfried et al. 2012; Kiebacher et al. 2023). However, cryophilic species depend on a stress‐tolerant strategy and are therefore weak competitors compared to many thermophilic species (Fescenko et al. 2020). Therefore, they can increasingly establish in disturbed areas and show higher cover there, as our results also showed. However, we found mostly the species of the species pool in the undisturbed surrounding, which indicates an influence of the soil seed bank or seed dispersal by wind.

The analysis of the cover of the life forms shows that in disturbed sites, the cover of dwarf shrubs and lichens was significantly lower. Due to their slow growth rates, these species face challenges in establishing themselves on unstable, rocky substrates (Sancho et al. 2001). Trees, dwarf shrubs, bryophytes and lichens are typically characteristic of more advanced succession stages and stable site conditions (Cray and Pollard 2015). This is consistent with our findings. Plants with higher growth rates are more resilient and possess a greater regenerative potential, enabling them to re‐colonise open areas more quickly and efficiently (Bernhardt‐Römermann et al. 2011; Cray and Pollard 2015). Re‐colonisation after disturbance primarily occurs through grasses and herbs. Our results confirm this as well: in disturbed sites, the cover of graminoids was around 30%, and that of herbs was 51%. Together, these two life forms account for about 81% of the vegetation in the disturbed areas. The re‐colonisation of the disturbed sites along the elevation gradient might also be influenced by the species available from the seed bank in the soil and the highly developed vegetation in the surrounding.

### 4.3 Effects of GMD on Phenotypic Plasticity

Adaptations in plasticity in response to GMDs in relation to plant ecological strategies and their relevance for morphological and physiological leaf traits, as well as in relation to the variability in different functional plant groups, are still largely unknown (Novakovskiy et al. 2016). We hypothesised that the high phenotypic plasticity of alpine plant species would result in shorter growth, smaller leaves and a reduction in SLA in disturbed areas. To test this, we measured important aboveground plant traits in the same species. We did this along elevation transects in disturbed and undisturbed plots and compared the responses along the transect.

From other studies, we knew that SLA was increased on degraded sites for R‐strategists (Novakovskiy et al. 2016), which was also shown by our results. Li et al. (2021) also found that above‐ and belowground biomass was decreased on severely degraded sites. However, these differences are likely to be largely due to altered site factors and the associated changes in growth conditions and not directly to the GMDs. Our results showed that disturbed areas had higher soil dispersion and light availability and lower humus and nutrient content. At the same time, plant height and leaf dry weight were decreased, whereas leaf area and SLA were increased. A decrease in plant height in grassland stands with lower light availability is a well‐known phenomenon (Chen et al. 2010; Siebenkäs et al. 2015), which explains the lower plant height in open, disturbed areas. The effects of shading on SLA, on the other hand, are not as clear, but there is evidence that, especially in grasses, rather than forbs, increasing shading tends to increase SLA. Thus, the increase in SLA cannot be explained by changing light conditions. However, the changed nutrient and humus conditions may be responsible for this. In many forb species, SLA tended to decline with increasing nutrient availability (Siebenkäs et al. 2015). We only measured above‐ground plant traits, but we know that GMD and the resulting changes in site conditions also have a massive impact on underground traits. For example, it is generally known that alpine plant species invest more in their root system over the course of their lifecycle than lowland species (Humphries 2020). The less favourable the above‐ground growing conditions are, the higher this proportion tends to be. Furthermore, the root system is increasingly spread out horizontally, with a large proportion of fine roots, especially in open, disturbed soil conditions (Pohl et al. 2011). This root system ensures that water and nutrients can be efficiently absorbed from the topsoil layer during the short growing season. At the same time, however, it also increases the aggregate stability of the topsoil, which in turn stabilises the soil and makes it less susceptible to erosion. However, due to a lack of underground trait measurements, we are unable to prove such relationships in this study.

### 4.4 Methodological Limitations

Some methodological approaches need to be critically discussed: The number of plots is not uniform in all three study areas, which introduces a bias into the study. The reason for this was twofold. Firstly, the elevation range of the transects differed between the study areas. Secondly, we could only use plot pairs that met the selection criteria (disturbed/undisturbed, not too far apart, identical site conditions). To account for this bias, the evaluations were carried out per area. Only then was the data analysed together. This was done so that area‐specific differences can be identified. Furthermore, the statistical methods used (GAMs) are robust against sampling bias, provided that the data set for each area contains more than 100 samples (see also, Gaul et al. 2020). The sufficiently high sample size per area increases the probability of successful model fitting, especially when accompanied by a reduction in spatial bias. In this study, spatial bias was minimised by the joint design of the surveys, a sufficiently high sample size (> 155 samples) and homogeneous selection criteria for the plots, which ensured the representativeness of all samples.

However, the temporal aspect of the disturbance could not be taken into account when selecting the plots. Unfortunately, no data on the temporal scale of the disturbance was available, so it could not be considered an explanatory factor.

## 5 Conclusion

In this study, we investigated differences in vegetation condition between undisturbed and disturbed sites along elevation transects in three alpine sites in the European Alps. The undisturbed and disturbed sites differed in plant community composition, total vegetation cover and species composition. The GMD did emerge as one important driver for plant community composition; however, climatic and edaphic variables played a more pronounced role. Our study showed that re‐colonisation after disturbance in alpine areas occurs mainly through herbs and graminoids. We were also able to demonstrate the effects of GMD on the plasticity of alpine plant species, although it has not been conclusively clarified whether phenotypic plasticity is controlled by changes in nutrient and humus conditions and light availability rather than by the disturbance itself. The results of this study thus contribute to a better understanding of vegetation development in an unstable and extreme habitat, that is, the harsh climate conditions. Albeit we only focused on the above‐ground functional plant traits in the study. In the future, it would also be useful to take a closer look at the changes in the root system in order to quantify the stabilising effect of a well‐developed root system as a counterpart to GMD. The mechanisms responsible for this have not yet been fully researched and offer opportunities for further research.

## Author Contributions

**Sarah Kinzner:** data curation (equal), formal analysis (equal), methodology (equal), visualization (equal), writing – original draft (equal), writing – review and editing (equal). **Katharina Ramskogler:** conceptualization (lead), data curation (equal), formal analysis (equal), funding acquisition (supporting), investigation (equal), methodology (equal), project administration (lead), supervision (supporting), visualization (equal), writing – original draft (equal), writing – review and editing (equal). **Sophia Castlunger:** data curation (equal), formal analysis (equal), investigation (equal), methodology (equal), writing – review and editing (equal). **Florentin Hofmeister:** data curation (supporting), writing – original draft (equal), writing – review and editing (equal). **Erich Tasser:** conceptualization (lead), formal analysis (equal), funding acquisition (lead), methodology (equal), project administration (lead), supervision (lead), writing – original draft (equal), writing – review and editing (equal).

## Funding

This work was supported by Autonomous Province of Bozen/Bolzano, South Tyrol, IT‐DFG 781607.

## Conflicts of Interest

The authors declare no conflicts of interest.

## Supporting information


**Data S1:** ece373056‐sup‐0001‐supinfo.docx.

## Data Availability

The trait are published in PANGEA (Ramskogler, Kinzner, et al. 2026). The environmental data for all plots are published in Ramskogler, Hofmeister, et al. (2026). The supporting information is available in dryad: Supporting information from: Influence of geomorphic disturbance on phenotypic plasticity and vegetation cover in high‐elevated belts: https://doi.org/10.5061/dryad.rjdfn2zs1.
