# Supplementary material for: Influence of Geomorphic Disturbance on Phenotypic Species Plasticity and Vegetation Cover in High‐Elevated Belts
Source: Ecol Evol. 2026 Mar 12;16(3):e73056. doi: 10.1002/ece3.73056 (PMC13093362; doi:10.1002/ece3.73056)
Supplement: Supplementary file 1 — Data S1: ece373056‐sup‐0001‐supinfo.docx. [file ECE3-16-e73056-s001.docx]

**Table S1:** Climate stations for the description of the study areas and the date of access. KA = Kauner Valley, HO = Horlach Valley, and MA = Martell Valley.

| study area | station | elevation of station (m a.s.l.) | date of access |
| --- | --- | --- | --- |
| KA | Feichten | 1314 | 5^th^ June 2023 |
| HO | WF Hailachbach | 1910 | 11^th^ February 2025 |
| MA | Zufrittt | 1851 | 21^st^ September 2022 |

**Table S2:** Characterisation of the study areas. KA = Kauner Valley, HO = Horlach Valley, MA = Martell Vallley, SPI = Stream Power Index, N = northness, E = eastness, mean temp = mean temperature over the last five years, precipitation = mean precipitation over the last five years.

| study area | elevation transect | type of disturbance | cover vascular species (%) | sampling frequency (n) | elevation (m a.s.l.) | SPI | aspect | N | E | inclination (°) | mean temp  (°C) | precipitation  (mm) |
| --- | --- | --- | --- | --- | --- | --- | --- | --- | --- | --- | --- | --- |
| KA | 5 | / | 87.0 | 2 | 2597 | 2.97 | W | -0.073 | -0.997 | 21.29 | -0.14 | 1133.11 |
| KA | 5 | Solifluktion | 70.0 | 2 | 2645 | 3.57 | SW | -0.730 | -0.684 | 35.50 | -0.20 | 1137.38 |
| KA | 6 | / | 75.0 | 2 | 2731 | 4.03 | W | -0.011 | -1.000 | 32.11 | -0.73 | 1147.01 |
| KA | 6 | Soil erosion | 55.0 | 2 | 2724 | 3.95 | SW | -0.457 | -0.890 | 33.07 | -0.63 | 1145.94 |
| KA | 7 | / | 62.0 | 2 | 2806 | 1.32 | NW | 0.460 | -0.888 | 20.43 | -1.11 | 1154.54 |
| KA | 7 | Soil erosion | 3.3 | 2 | 2801 | 3.69 | W | -0.376 | -0.927 | 23.91 | -1.04 | 1153.89 |
| HO | 1 | / | 88.0 | 1 | 2118 | 2.62 | SW | -0.746 | -0.666 | 34.38 | 1.68 | 951.61 |
| HO | 1 | Soil erosion | 45.0 | 1 | 2121 | 2.05 | SW | -0.391 | -0.920 | 37.78 | 1.70 | 950.97 |
| HO | 4 | / | 71.0 | 1 | 2469 | 4.13 | W | -0.257 | -0.967 | 25.53 | -0.59 | 986.66 |
| HO | 4 | Soil erosion | 0.61 | 1 | 2464 | 5.22 | W | -0.302 | -0.953 | 30.05 | -0.45 | 984.85 |
| HO | 5 | / | 89.0 | 1 | 2579 | 3.11 | SE | -0.750 | 0.661 | 36.76 | -0.88 | 994.59 |
| HO | 5 | Soil erosion | 6.0 | 1 | 2562 | 4.96 | S | -0.948 | 0.320 | 21.62 | -0.93 | 995.64 |
| HO | 6 | / | 93.5 | 1 | 2648 | 2.04 | S | -0.938 | 0.346 | 37.57 | -1.28 | 1001.95 |
| HO | 6 | Soil erosion | 18.0 | 1 | 2643 | 4.02 | S | -0.994 | 0.106 | 34.83 | -1.85 | 1011.40 |
| HO | 7 | / | 88.0 | 1 | 2736 | 1.66 | SE | -0.918 | 0.396 | 27.72 | -1.85 | 1011.51 |
| HO | 7 | Rockfall | 4.5 | 1 | 2737 | 4.28 | S | -0.992 | 0.126 | 27.40 | -1.28 | 1001.95 |
| MA | 1 | / | 88.0 | 2 | 2207 | 5.48 | E | -0.033 | 0.999 | 23.26 | 2.44 | 1148.45 |
| MA | 1 | Soil erosion | 37.5 | 2 | 2220 | 3.66 | SE | -0.565 | 0.825 | 44.20 | 2.38 | 1149.62 |
| MA | 2 | / | 75.0 | 2 | 2354 | 3.05 | NE | 0.627 | 0.779 | 35.01 | 1.09 | 1173.75 |
| MA | 2 | Rockfall | 9.0 | 2 | 2367 | 4.68 | NE | 0.912 | 0.410 | 57.07 | 0.91 | 1173.01 |
| MA | 3 | / | 77.0 | 2 | 2478 | 6.52 | SE | -0.899 | 0.438 | 28.93 | 1.16 | 1207.29 |
| MA | 3 | Rockfall / Avalanche | 60.0 | 2 | 2477 | 2.65 | SE | -0.685 | 0.728 | 25.32 | 1.16 | 1208.33 |
| MA | 4 | / | 90.5 | 2 | 2507 | 1.76 | SE | -0.884 | 0.468 | 16.28 | 1.00 | 1221.32 |
| MA | 4 | Rockfall | 45.0 | 2 | 2518 | 8.07 | SE | -0.908 | 0.418 | 23.92 | 0.96 | 1223.02 |
| MA | 5 | / | 92.5 | 2 | 2645 | 1.12 | S | -0.990 | -0.140 | 17.05 | 0.25 | 1252.62 |
| MA | 5 | Avalanche | 41.0 | 2 | 2654 | 1.92 | S | -0.989 | -0.146 | 18.87 | 0.17 | 1253.07 |
| MA | 6 | / | 86.0 | 2 | 2778 | 0.67 | SE | -0.590 | 0.807 | 11.09 | -0.58 | 1264.71 |
| MA | 6 | Soil erosion | 30.0 | 2 | 2778 | 3.74 | E | -0.073 | 0.997 | 9.20 | -0.58 | 1264.71 |
| MA | 7 | / | 66.5 | 2 | 2854 | 3.32 | S | -0.963 | -0.268 | 24.84 | -0.96 | 1306.56 |
| MA | 7 | Soil erosion | 2.0 | 2 | 2857 | 3.10 | S | -0.999 | -0.054 | 28.98 | -0.96 | 1307.04 |

**Table S3**: Metadata of meteorological stations from Kauner Valley.

| **Station** | **Elevation (m a.s.l)** | **Variables** | **UTM East** | **UTM North** | **Provider** |
| --- | --- | --- | --- | --- | --- |
| Dammfuss | 1770 | T, P | 632260.068 | 5202225.11 | TIWAG |
| Gepatschalm | 1900 | T, P | 632251.149 | 5195058.38 | TIWAG |
| Weisssee | 2540 | T, P | 630648.401 | 5192468.24 | TIWAG |
| Fagge | 2095 | T | 632652.806 | 5193411.55 | Uni Bremen |
| Rifflsee Talstation Grubenkopf | 2227 | T | 640894.889 | 5202717.1 | LWD |

Meaning of the abbreviations:

T = temperature, P = precipitation, TIWAG = TIWAG-Tiroler Wasserkraft AG, LWD = Lawinenwarndienst Tirol.

**Table S4**: Metadata of meteorological stations from Horlach Valley.

| **Station** | **Elevation (m a.s.l)** | **Variable** | **East** | **North** | **Provider** |
| --- | --- | --- | --- | --- | --- |
| Leiter | 1550 | T, P | 649354.093 | 5220696.08 | TIWAG |
| Kraftwerk Kuehtai | 1970 | T, P | 651906.592 | 5230134.91 | TIWAG |
| Horlachalm | 1910 | T, P | 652550.376 | 5224655.94 | TIWAG |
| Umhausen | 1035 | T, P | 646261.656 | 5222434.37 | Geosphere |
| Grastal | 1951 | T | 651119.464 | 5221344.62 | Uni Bremen |
| Breiter Grieskogel Schneestation | 2385 | T | 653859.862 | 5216492.38 | LWD |
| Breiter Grieskogel Windstation | 3282 | T | 653507.375 | 5218373.61 | LWD |

**Table S5**: Metadata of meteorological stations from Martell Valley.

| **Station** | **Elevation (m a.s.l)** | **Variable** | **East** | **North** | **Provider** |
| --- | --- | --- | --- | --- | --- |
| Schoentaufspitze | 3328 | T | 624957 | 5151212 | Bolzano |
| Madritsch | 2825 | T | 623901 | 5150187 | Bolzano |
| Sulden | 1907 | T, P | 622375 | 5152632 | Bolzano |
| Hintermartell | 1720 | T, P | 632465 | 5152929 | Bolzano |
| Weissbrunnspitz | 3253 | T | 636135 | 5150463 | Bolzano |
| Rossbaenke | 2255 | T | 639686 | 5147806 | Bolzano |
| Weissbrunn | 1900 | T, P | 640591 | 5149772 | Bolzano |
| Careser | 2600 | T, P | 630560 | 5142404 | Trentino |
| Ghiacciaio | 3093 | T | 631963 | 5145624 | Trentino |
| Langenferner | 2967 | T | 623900.1 | 5147807 | UIBK |

Meaning of the abbreviations:

Bolzano = Provincia autonoma di Bolzano - Alto Adige, Trentino = Meteotrentino, UIBK = Universität Innsbruck.

**Table S6:** Site characterisation of transect plots based on the community-weighted means (CMW) of the different Landolt indicator values (Landolt et al., 2010). KA = Kauner Valley, HO = Horlach Valley, MA = Martell Vallley), light availability (L), temperature (T), soil conditions like humus (H), nutrient content (N), soil reactivity (R), soil dispersion (D), and soil moisture (F).

| study area | elevation transect | | type of disturbance | L | T | H | N | R | D | F |
| --- | --- | --- | --- | --- | --- | --- | --- | --- | --- | --- |
| KA | 5 | / | | 4.11 | 1.76 | 3.40 | 2.05 | 1.96 | 2.57 | 2.64 |
| KA | 5 | Solifluktion | | 4.25 | 1.62 | 3.15 | 2.08 | 2.08 | 2.99 | 2.44 |
| KA | 6 | / | | 4.41 | 1.45 | 3.38 | 1.96 | 2.06 | 2.33 | 2.69 |
| KA | 6 | Soil erosion | | 4.64 | 1.28 | 3.10 | 1.92 | 1.89 | 3.10 | 2.56 |
| KA | 7 | / | | 4.65 | 1.22 | 3.04 | 1.91 | 1.94 | 2.73 | 2.56 |
| KA | 7 | Soil erosion | | 4.75 | 1.22 | 2.88 | 2.06 | 2.31 | 2.39 | 3.00 |
| HO | 1 | / | | 3.33 | 2.08 | 4.34 | 2.02 | 1.98 | 2.52 | 2.75 |
| HO | 1 | Soil erosion | | 3.61 | 2.24 | 4.00 | 2.05 | 1.95 | 2.32 | 2.76 |
| HO | 4 | / | | 3.72 | 1.80 | 3.89 | 2.12 | 1.99 | 2.39 | 2.86 |
| HO | 4 | Soil erosion | | 4.33 | 1.49 | 3.63 | 1.71 | 1.70 | 3.61 | 2.37 |
| HO | 5 | / | | 4.12 | 1.80 | 3.25 | 2.10 | 2.38 | 2.79 | 2.73 |
| HO | 5 | Soil erosion | | 4.31 | 1.48 | 3.00 | 2.17 | 2.17 | 3.27 | 2.90 |
| HO | 6 | / | | 3.87 | 1.58 | 3.74 | 2.03 | 1.88 | 2.38 | 2.80 |
| HO | 6 | Soil erosion | | 4.55 | 1.33 | 3.16 | 2.08 | 2.40 | 2.08 | 2.93 |
| HO | 7 | / | | 4.29 | 1.41 | 3.26 | 2.18 | 2.36 | 2.81 | 2.91 |
| HO | 7 | Rockfall | | 4.59 | 1.27 | 3.00 | 2.14 | 2.36 | 3.18 | 3.11 |
| MA | 1 | / | | 2.88 | 2.27 | 4.50 | 2.05 | 1.67 | 1.99 | 3.01 |
| MA | 1 | Soil erosion | | 4.47 | 1.43 | 2.61 | 2.11 | 2.16 | 3.88 | 2.93 |
| MA | 2 | / | | 3.67 | 1.75 | 4.04 | 1.99 | 1.82 | 2.11 | 2.79 |
| MA | 2 | Rockfall | | 4.47 | 1.43 | 2.61 | 2.11 | 2.16 | 3.88 | 2.93 |
| MA | 3 | / | | 3.63 | 1.92 | 3.27 | 2.08 | 2.53 | 3.17 | 2.28 |
| MA | 3 | Rockfall / Avalanche | | 3.69 | 1.89 | 3.43 | 2.18 | 2.39 | 2.94 | 2.46 |
| MA | 4 | / | | 4.21 | 1.73 | 2.87 | 2.00 | 3.24 | 3.55 | 2.22 |
| MA | 4 | Rockfall | | 4.18 | 1.62 | 2.76 | 2.04 | 3.47 | 3.59 | 2.44 |
| MA | 5 | / | | 4.22 | 1.67 | 3.04 | 2.01 | 2.43 | 3.04 | 2.57 |
| MA | 5 | Avalanche | | 4.39 | 1.47 | 3.13 | 2.15 | 2.27 | 2.74 | 2.90 |
| MA | 6 | / | | 4.71 | 1.27 | 3.03 | 1.80 | 2.20 | 3.24 | 2.33 |
| MA | 6 | Soil erosion | | 4.64 | 1.24 | 3.19 | 2.08 | 2.09 | 2.50 | 2.81 |
| MA | 7 | / | | 4.67 | 1.18 | 2.85 | 1.99 | 2.22 | 3.08 | 2.83 |
| MA | 7 | Soil erosion | | 4.83 | 1.13 | 2.34 | 1.83 | 2.17 | 3.51 | 3.03 |

**Table S7**: Species used for the trait analysis with the according plot pairs (no. = number, KA = Kauner Valley, HO = Horlach Valley, MA = Martell Valley), the number beside the valley code indicates the elevation.

| **no.** | **species** | **plot pair** | **no.** | **species** | **plot pair** |
| --- | --- | --- | --- | --- | --- |
| **1** | *Agrostis rupestris* | MA7 | **15** | *Leucanthemopsis alpina* | KA6/7, HO6/7, MA5 |
| **2** | *Anthoxanthum odoratum* agg. | HO7, MA2 | **16** | *Oreochloa disticha* | KA6 |
| **3** | *Arctostaphylos uva-ursi* | MA4 | **17** | *Phyteuma hemisphaericum* | HO5 |
| **4** | *Avenella flexuosa* | HO1, MA2 | **18** | *Priumula glutinosa* | MA6 |
| **5** | *Cardamine resedifolia* | MA7 | **19** | *Rhododendron ferrugineum* | HO4 |
| **6** | *Carex curvula* | KA5/7, HO4/5, MA6 | **20** | *Salix herbacea* | KA7 |
| **7** | *Daphne striata* | MA3/4 | **21** | *Salix serpilifolia* | MA4 |
| **8** | *Festuca halleri* | MA6 | **22** | *Saxifraga byroides* | MA7 |
| **9** | *Geum montanum* | MA5 | **23** | *Saxifraga paniculata* | MA1 |
| **10** | *Gnaphalium supinum* | HO7 | **24** | *Silene rupestris* | HO1 |
| **11** | *Helictochloa versicolor* | KA5/6 | **25** | *Thymus praecox* ssp*. polytrichus* | MA3 |
| **12** | *Jacobaea incanca* | HO6 | **26** | *Trifolium alpinum* | KA5 |
| **13** | *Juncus trifidus* | HO4/5/6, MA2 | **27** | *Vaccinium vitis-idaea* | MA1 |
| **14** | *Juniperus communis var. saxatilis* | MA1/3 | **28** | *Veronica bellidioides* | MA5 |

**Data S1**

The 10 environmental variables were reduced to three rotated components (RC1–RC3). The three components explained 81 % of the variance. RC1 accounted for 39 % of the variance, RC2 for 21 %, and RC3 for 21 %, respectively (**Table S8a**). RC1 included, among others, Landolt indicator value T (0.96), elevation (-0.87), and mean annual temperature (0.74; **Table S8b**). Therefore, RC1 summarised key elevation-related climate parameters, whereby increasing component values mean improved growth conditions. RC2 included primarily edaphic parameters such as nutrient availability (0.85), continentality (-0.76) and water availability in the soil (0.75; **Table S8b**). Increasing values of the components thus reflect improved edaphic conditions. RC3 was positive with pH (0.86) and dispersity (0.72) and negative with humus (-0.67; **Table S8b**). This component will be referred to as less acidic debris sites. The variables SPI, northness, eastness, slope and precipitation were separately used in the GAM as they did not load sufficiently in the components RC1 to RC3.

**Table S8**: a) Standardised loadings with the proportion explained for the three rotated components (RC) and b) the loadings of each variable to the components; cwm = community weighted mean, LIV = Landolt indicator value.

| **a)** | **RC1** | **RC2** | **RC3** |
| --- | --- | --- | --- |
| SS loadings | 3.90 | 2.07 | 2.14 |
| Proportion Var | 0.39 | 0.21 | 0.21 |
| Proportion Explained | 0.48 | 0.26 | 0.26 |
|  |  |  |  |
| **b)** | **RC1** | **RC3** | **RC2** |
| cwm LIV temperature | 0.96 |  |  |
| cwm LIV light | -0.88 |  |  |
| elevation | -0.87 |  |  |
| 5-years mean mean annual temperature | 0.74 |  |  |
| cwm LIV humus | 0.67 | -0.67 |  |
| cwm LIV soil reaction |  | 0.86 |  |
| cwm LIV dispersity |  | 0.72 |  |
| cwm LIV nutrients |  |  | 0.85 |
| cwm LIV continentality |  |  | -0.76 |
| cwm LIV moisture |  |  | 0.75 |

The equations of the generalised additive models (GAMs) of the final models for each response variable are the following:

$g\left( E\left[ total cover \right] \right)= \beta_{0}+ \beta_{1}\text{"}\text{climat}\text{ induced growth}\text{"}+ \beta_{2}\text{"}\text{improved edaphic conditions}\text{"}+ f_{1}\left( \text{less acidic debris} \right)+ \beta_{3}SPI+ \beta_{4}Northness+ \beta_{5}Eastness+ \beta_{6}slope+ f_{2}\left( sum precipitation \right)+ \sum_{k=1}^{K-1} \beta_{k}GMD$ **Eq. 1**

$g\left( E\left[ species richness \right] \right)= \beta_{0}+ \beta_{1}\text{climate induced growth}+ \beta_{2}\text{improved edaphic conditions}+ f_{1}\left( \text{less acidic debris} \right)+ \beta_{3}SPI+ f_{2}\left( northness \right)+ f_{3}\left( eastness \right)+ \beta_{4} slope+ \beta_{5} sum precipitation+ \sum_{k=1}^{K-1} \beta_{k}GMD$ **Eq. 2**

$g(E\left[ rel. cov. cryophilic sp. \right)))= \beta_{0}+ \beta_{1}"\text{climate induced growth}" + f_{1}"improved edaphic conditions"+ \beta_{2}"less acidic debris" + f_{2}(SPI)+ \beta_{3}northness+ \beta_{4}eastness+ \beta_{5}slope+ f_{3}(sum precipitation)+ \sum_{k=1}^{K-1} \beta_{k}GMD$ **Eq. 3**

$g\left( E\left[ rel. cov. thermophilic sp. \right] \right)= \beta_{0}+ \beta_{1}\text{"}\text{climate induced growth}\text{"}+ \beta_{2}\text{"}\text{improved edaphic conditions}\text{"}+ \beta_{3}\text{less acidic debris}+ f_{1}\left( SPI \right)+ \beta_{4}northness+ \beta_{52}+ \beta_{6}slope+ \beta_{74}sum precipitation+ \sum_{k=1}^{K-1} \beta_{k}GMD$ **Eq. 4**

$g\left( E\left[ rel. cov. competitive sp. \right] \right)= \beta_{0}+ \beta_{1}\text{"}\text{climate induced growth}\text{"}+ \beta_{2}\text{ "}\text{improved edaphic conditions}\text{"}+ \beta_{3}\text{"}\text{less acidic debris}\text{"}+ \beta_{34}SPI+ \beta_{5}norhtness+ f_{1}\left( eastness \right)+ \beta_{6}slope+ f_{2}\left( sum precipitation \right)+\sum_{k=1}^{K-1} \beta_{k}GMD$ **Eq. 5**

$g\left( E\left[ rel. cov. ruderal sp. \right] \right)= \beta_{0}+ f_{1}\text{(}\text{"}\text{climate induced growth}\text{"}\text{)}+ \beta_{1}\text{improved edaphic conditions}+ f_{2}\text{(}\text{"}\text{less acidic debris}\text{"}\text{)}+ f_{3}\left( SPI \right)+ \beta_{23}northness+ \beta_{3}eastness+ f_{4}\left( slope \right)+ \beta_{4}sum precipitation+\sum_{k=1}^{K-1} \beta_{k}GMD$ **Eq. 6**

$g\left( E\left[ \text{rel. cov. stress-tolerant sp.} \right] \right)= \beta_{0}+ \beta_{1}\text{"}\text{climate induced growth}\text{"}+ \beta_{2}\text{ "}\text{improved edaphic conditions}\text{" + }\text{β}_{\text{3}}\text{less acidic debris}+ \beta_{4}SPI+ \beta_{5}northness+ f_{1}\left( eastness \right)+ \beta_{6}slope+ \beta_{6}sum precipitation+\sum_{k=1}^{K-1} \beta_{k}GMD$ **Eq. 7**

$g\left( E\left[ \text{rel. cov. csr-strategists} \right] \right)= \beta_{0}+ \beta_{1}\text{"}\text{climate induced growth}\text{"}+ \beta_{2}\text{improved edaphic conditions}+ \beta_{3}\text{"}\text{less acidic debris}\text{"}+ f_{1}\left( SPI \right)+ \beta_{4}northness+ f_{2}\left( eastness \right)+ \beta_{5}slope+ \beta_{6}sum precipitation+\sum_{k=1}^{K-1} \beta_{k}GMD$ **Eq. 8**

$g\left( E\left[ rel. cov. bryophytes \right] \right)= \beta_{0}+ f_{1}\left( \text{"}\text{climate induced growth}\text{"} \right)+ \beta_{1}\text{"}\text{improved edaphic conditions}\text{"}+ \beta_{2}\text{"}\text{less acidic debris}\text{"}+ f_{2}\left( SPI \right)+ f_{3}\left( northness \right)+ f_{4}\left( eastness \right)+ \beta_{3}slope+ \beta_{4}sum precipitation+\sum_{k=1}^{K-1} \beta_{k}GMD$ **Eq. 9**

$g\left( E\left[ rel. cov. dwarf shrubs \right] \right)= \beta_{0}+ \beta_{1}\text{"}\text{climate induced growth}\text{"}+ \beta_{2}\text{"}\text{improved edaphic conditions}\text{"}+ f_{1}\left( \text{"}\text{less acidic debris}\text{"} \right)+ \beta_{3}SPI+ f_{2}\left( northness \right)+ f_{3}\left( eastness \right)+ \beta_{4}slope+ f_{4}\left( sum precipitation \right)+\sum_{k=1}^{K-1} \beta_{k}GMD$ **Eq. 10**

$g\left( E\left[ rel. cov. graminoids \right] \right)= \beta_{0}+ f_{1}\left( \text{"}\text{climate induced growth}\text{"} \right)+ f_{2}\text{(}\text{"}\text{improved edaphic conditions}\text{"}\text{)}+ \beta_{1}\text{less acidic debris}+ f_{3}\left( SPI \right)+ \beta_{2}northness+\beta_{3}eastness+ \beta_{4}slope+ \beta_{5}sum precipitation+\sum_{k=1}^{K-1} \beta_{k}GMD$ **Eq. 11**

$g\left( E\left[ rel. cov. lichens \right] \right)= \beta_{0}+ \beta_{1}\text{"}\text{climate induced growth}\text{"}+ \beta_{2}\text{"}\text{improved edaphic conditions}\text{"}+ \beta_{3}\text{"}\text{less acidic debris}\text{"}+ \beta_{4}SPI+ \beta_{5}northness+ \beta_{6}eastness+ \beta_{7}slope+ \beta_{8}sum precipitation+\sum_{k=1}^{K-1} \beta_{k}GMD$ **Eq. 12**

$g\left( E\left[ rel. cov. trees \right] \right)= \beta_{0}+ \beta_{1}\text{"}\text{climate induced growth}\text{"}+ \beta_{2}\text{"}\text{improved edaphic conditions}\text{" + }\text{β}_{\text{3}}\text{less acidic debris}+ \beta_{4}SPI+ f_{1}\left( northness \right)+ f_{2}\left( eastness \right)+ \text{β}_{\text{4}}\left( slope \right)+ f_{3}sum precipitation+ \sum_{k=1}^{K-1} \beta_{k}GMD$ **Eq. 13**

$g\left( E\left[ rel. cov. herbs \right] \right)= \beta_{0}+ \beta_{1}\text{"}\text{climate induced growth}\text{"}+ \beta_{2}\text{"}\text{improved edaphic conditions}\text{"}+ \beta_{3}\text{"}\text{less acidic debris}\text{"}+ f_{1}(SPI)+ f_{2}(northness)+ f_{3}(eastness)+ \beta_{4}slope+ \beta_{5}sum precipitation+ \sum_{k=1}^{K-1} \beta_{k}GMD$ **Eq. 14**

$g\left( E\left[ plant height \right] \right)= \beta_{0}+ \gamma_{0i} + f_{1}(\text{"}\text{climate induced growth}\text{") + }\beta_{1}\text{"}\text{improved edaphic conditions}\text{" + }f_{2}\text{(" }\text{less acidic debris}\text{") + }\beta_{2}\text{SPI}\text{ + }\beta_{3}\text{ northness}\text{ + }\beta_{4}\text{eastness}\text{ + }\beta_{5}\text{slope}\text{ + }f_{3}\text{(}\text{sum precipitation}\text{) + }\sum_{k=1}^{K-1} \beta_{k}lifeform\text{ + }\sum_{k=1}^{K-1} \beta_{k}GMD+\text{ }u_{species (i)}$ **Eq. 15**

$g\left( E\left[ log(leaf dry weight) \right] \right)= \beta_{0}+ \gamma_{0i} + \beta_{1}\text{"}\text{climate induced growth}\text{" + }\beta_{2}\text{"}\text{improved edaphic conditions}\text{" + }\beta_{3}\text{" }\text{less acidic debris}\text{" + }f_{1}\text{(SPI}\text{ )+ }\beta_{4}\text{ northness}\text{ + }\beta_{5}\text{eastness}\text{ + }f_{2}\text{(slope)}\text{ + }f_{3}\text{(}\text{sum precipitation}\text{) + }\sum_{k=1}^{K-1} \beta_{k}slifeform\text{ + }\sum_{k=1}^{K-1} \beta_{k}GMD+\text{ }u_{species (i)}$ **Eq. 16**

$g\left( E\left[ log(leaf area \right] \right)= \beta_{0}+ \gamma_{0i} + \beta_{1}\text{"}\text{climate induced growth}\text{" + }\beta_{2}\text{"}\text{improved edaphic conditions}\text{" + }\beta_{3}\text{" }\text{less acidic debris}\text{" + }\beta_{4}\text{SPI+ }f_{1}\text{(northness}\text{ ) + }\beta_{4}\text{eastness}\text{ + }f_{2}\text{(slope}\text{ )+ }f_{3}\text{(}\text{sum precipitation}\text{) + }\sum_{k=1}^{K-1} \beta_{k}lifeform\text{+}\sum_{k=1}^{K-1} \beta_{k}GMD+\text{ }u_{species (i)}$ **Eq. 17**

$g\left( E\left[ log(SLA) \right] \right)= \beta_{0}+ \gamma_{0i} + \beta_{1}\text{"}\text{climate induced growth}\text{" + }\beta_{2}\text{"}\text{improved edaphic conditions}\text{" + }f_{1}\text{(" }\text{less acidic debris}\text{") + }\beta_{3}\text{SPI}\text{ + }\beta_{4}\text{ northness}\text{ + }f_{2}\text{(eastness}\text{ )+ }f_{3}\text{(slope}\text{ )+ }f_{4}\text{(}\text{sum precipitation}\text{) + }\sum_{k=1}^{K-1} \beta_{k}lifeform\text{ + }\sum_{k=1}^{K-1} \beta_{k}GMD+\text{ }u_{species (i)}$ **Eq. 18**

where $g()$ is the link function and $E$ the expectation, total cover and rel. cov. (relative cover) the transformed ratio of the cover values, $\beta_{0}$ the overall intercept, $u_{species}\sim N(0, \sigma_{species}^{2})$ the normally distributed random intercept for species , $\beta_{i}$ the intercept, $f_{i}$ the smooth function, and *GMD* geomorphic disturbance. For the analyses of the transformed ratio of the cover values, the GAM model was fitted using a beta distribution and, for species richness, a Poisson distribution.


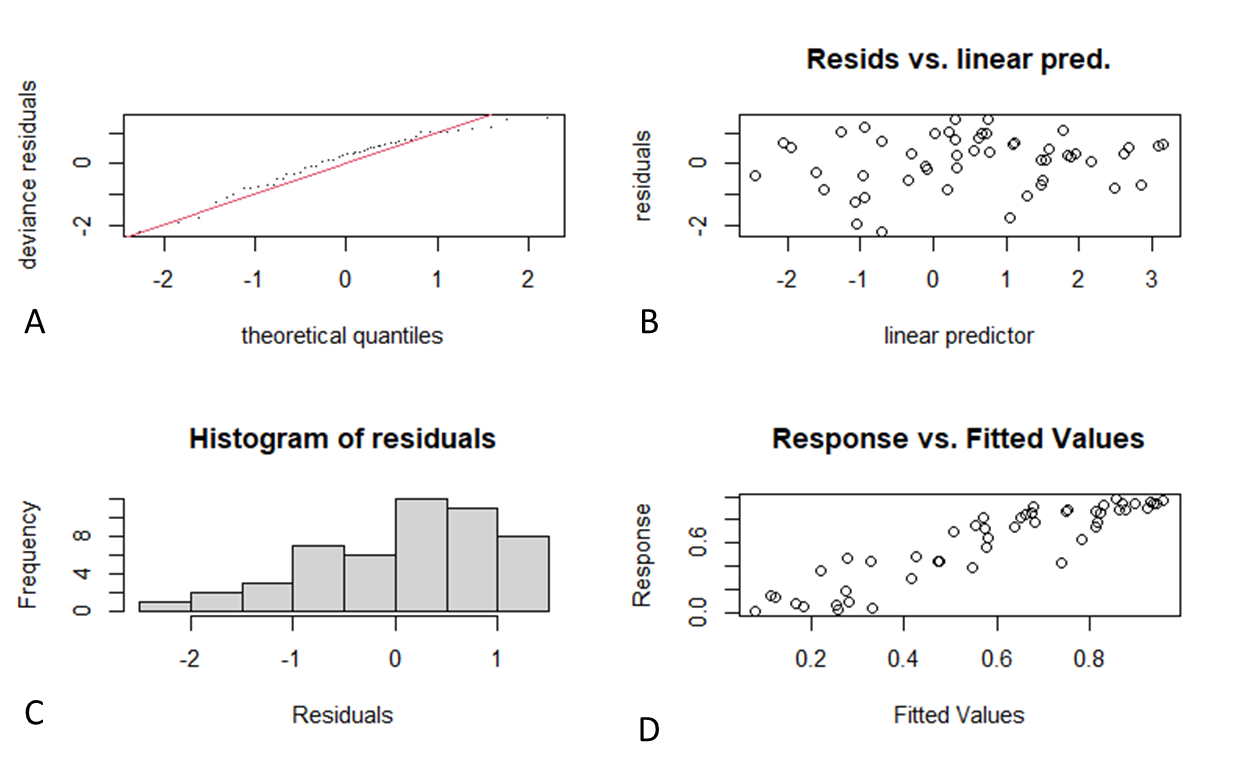


**Fig. S1**: Model’s diagnostics for the total cover response variable. A Quantile plot, B residuals vs. linear predictor, C distribution of residuals, and D observed vs. predicted values.


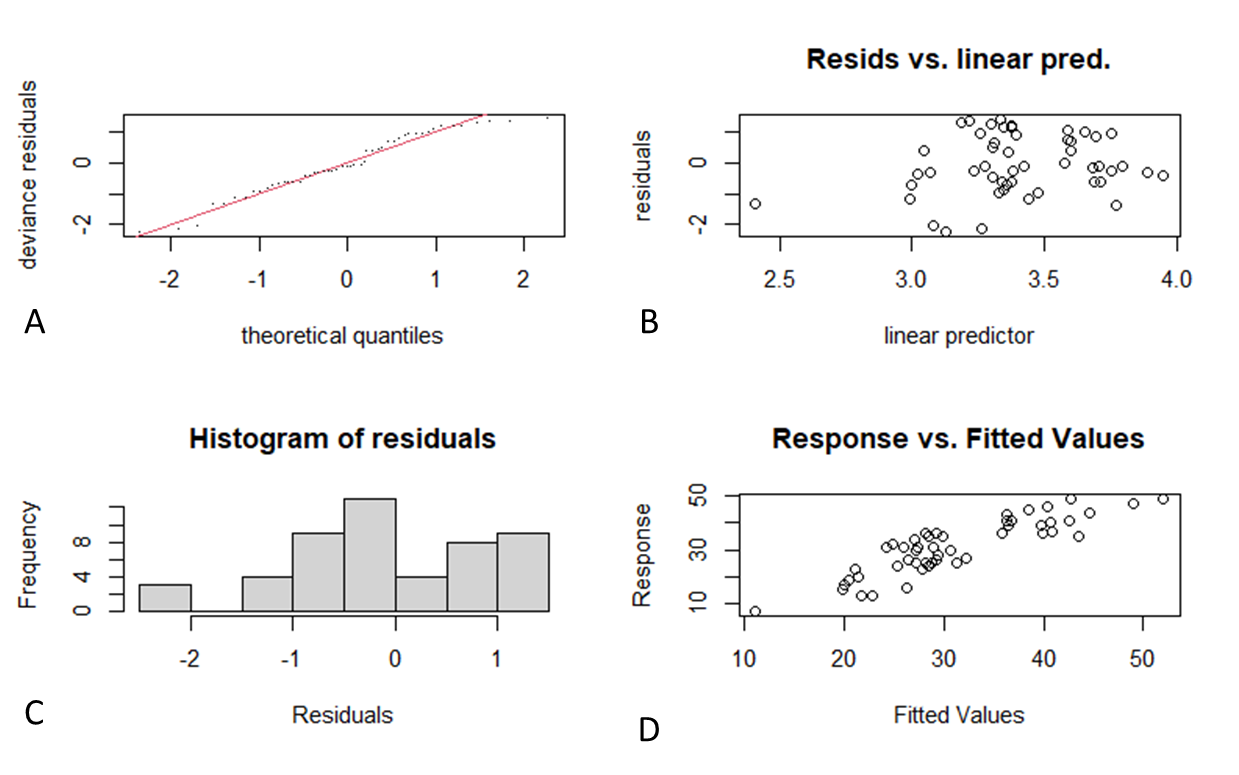


**Fig. S2**: Model’s diagnostic for the species richness response variable. A Quantile plot, B residuals vs. linear predictor, C distribution of residuals, and D observed vs. predicted values.


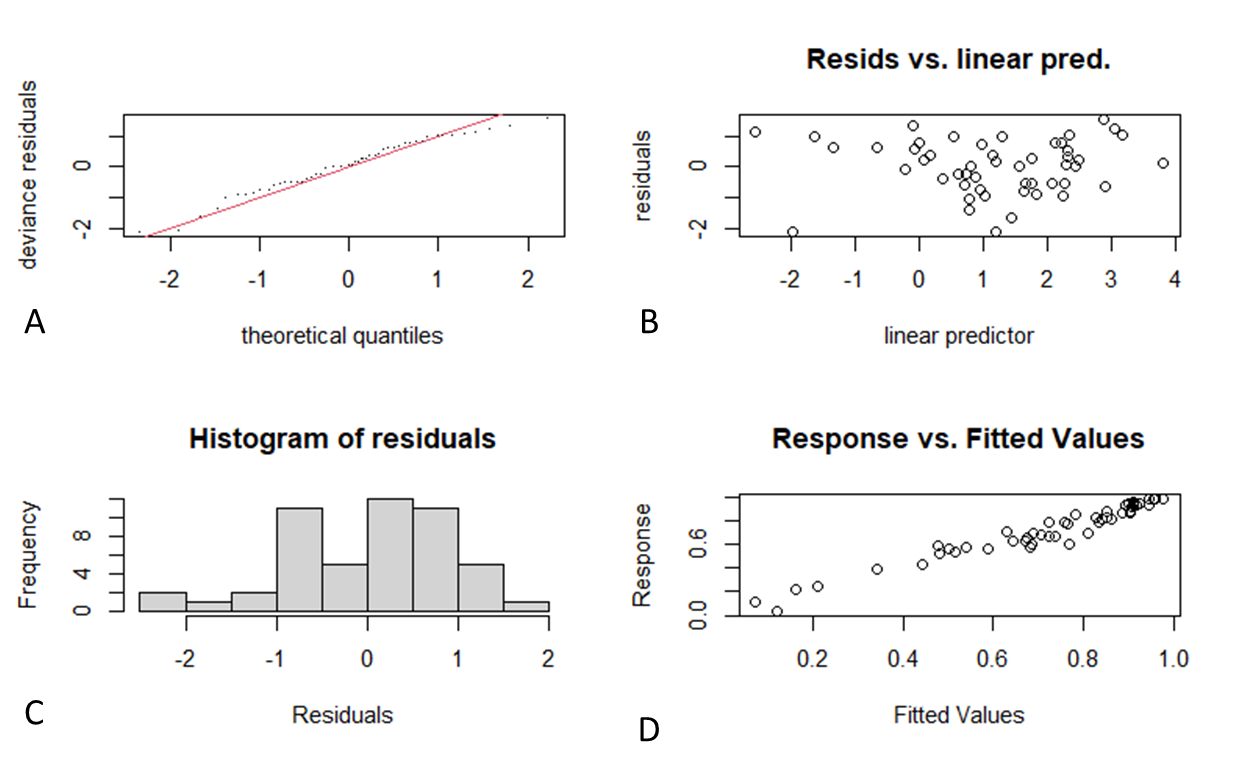


**Fig. S3**: Model’s diagnostic for the relative cover of cryophilic species response variable. A Quantile plot, B residuals vs. linear predictor, C distribution of residuals, and D response vs. predicted values.


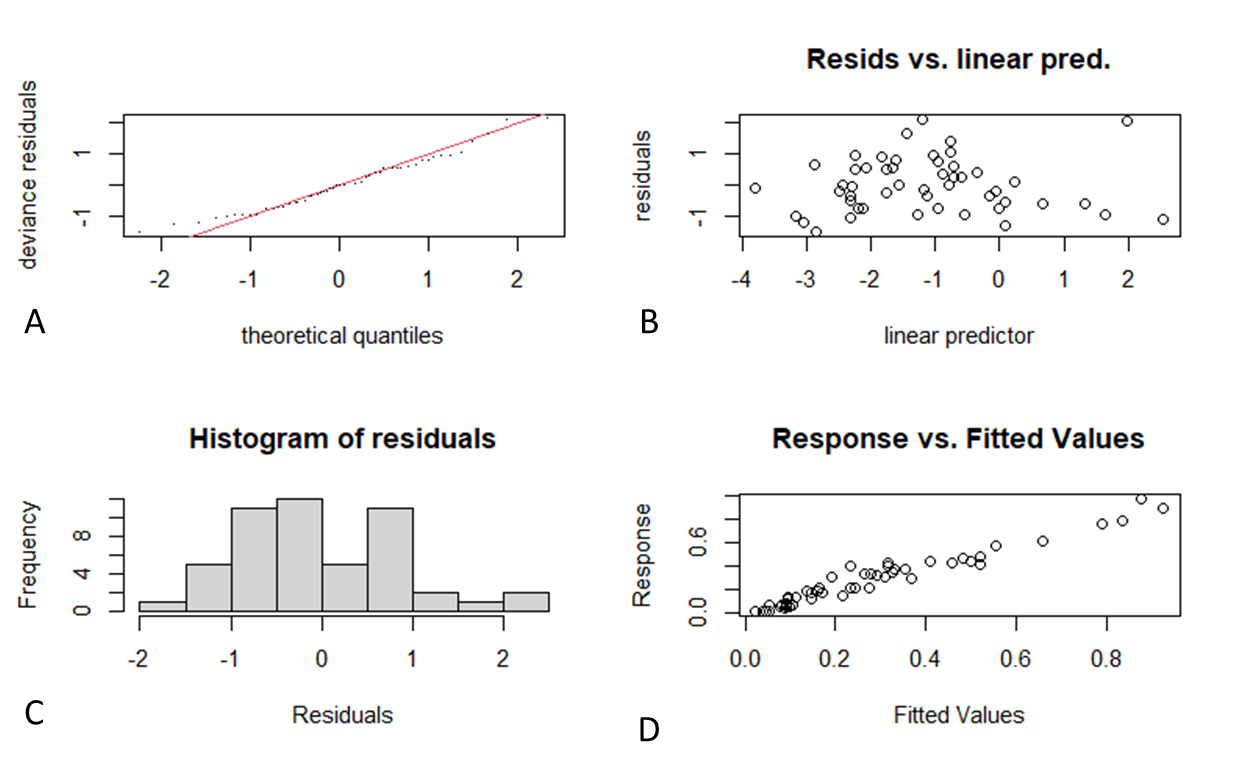


**Fig. S4**: Model’s diagnostic for the relative cover of thermophilic species response variable. A Quantile plot, B residuals vs. linear predictor, C distributions of residuals, and D response vs. predicted values.


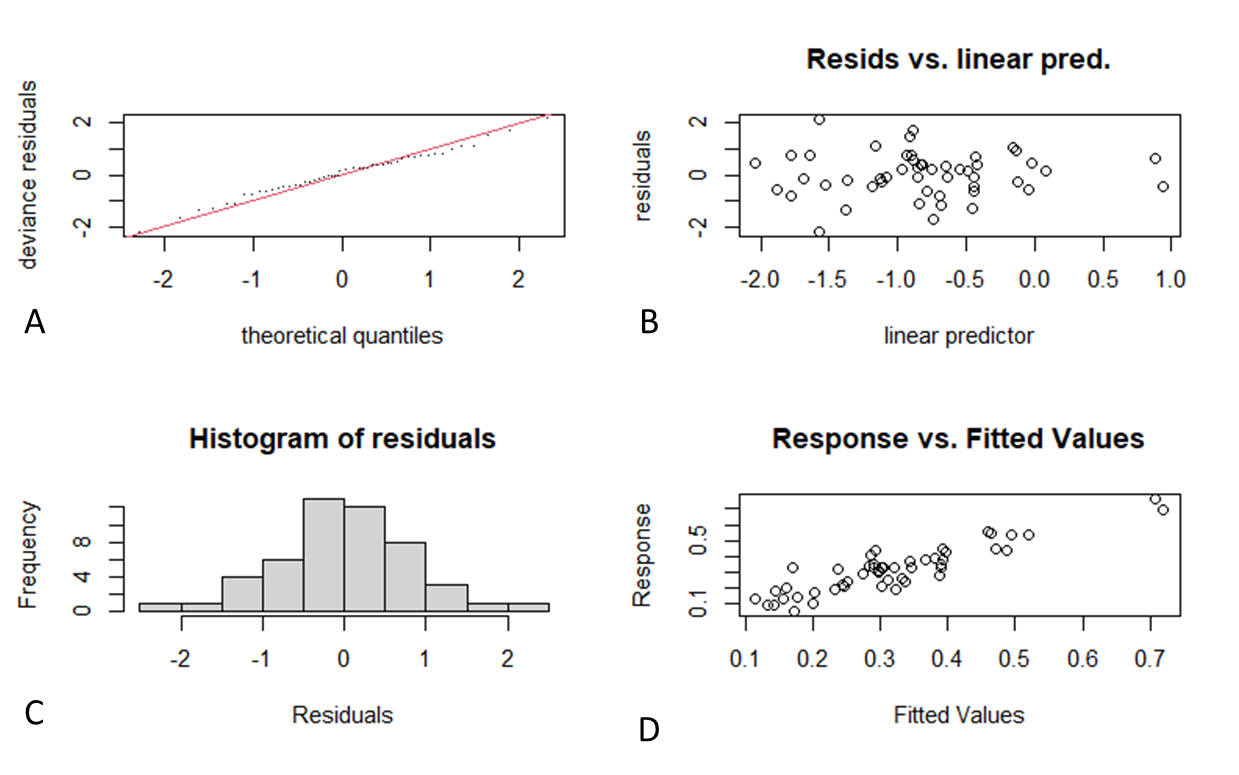


**Fig. S5**: Model’s diagnostic for the relative cover of competitive species response variable. A Quantile plot, B residuals vs. linear predictor, C distribution of residuals, and D response vs. predicted values.


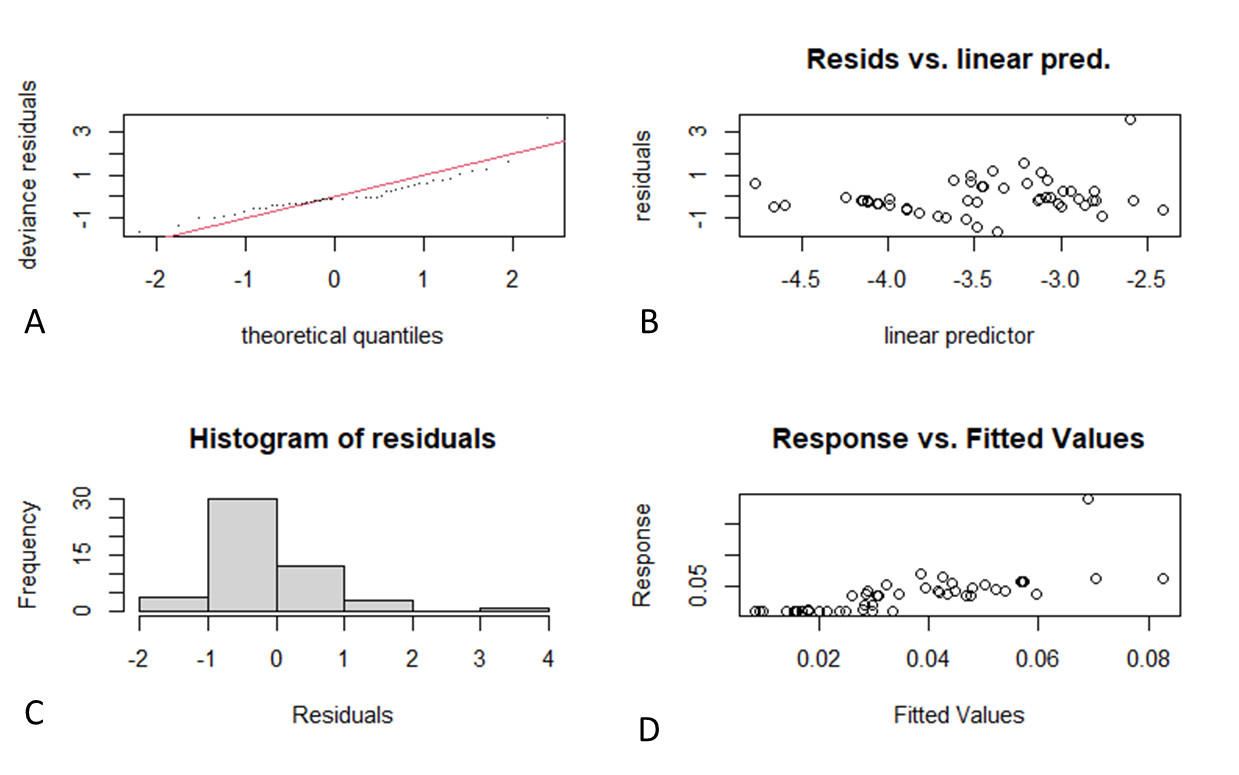


**Fig. S6**: Model’s diagnostic for the relative cover of ruderal species response variable. A Quantile plot, B residuals vs. linear predictor, C distribution of residuals, and D response vs. predicted values.


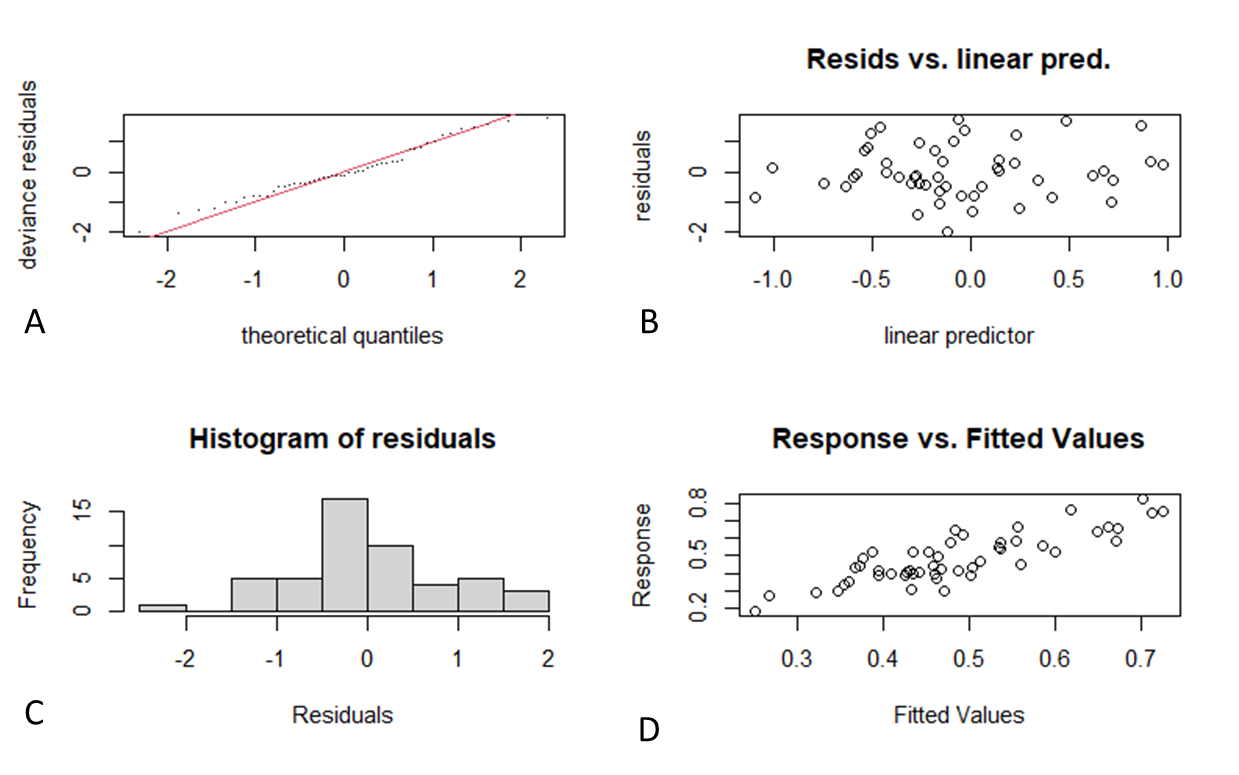


**Fig. S7**: Model’s diagnostic for the relative cover of stress-tolerant species response variable. A Quantile plot, B residuals vs. linear predictor, C distribution of residuals, and D response vs. linear values.


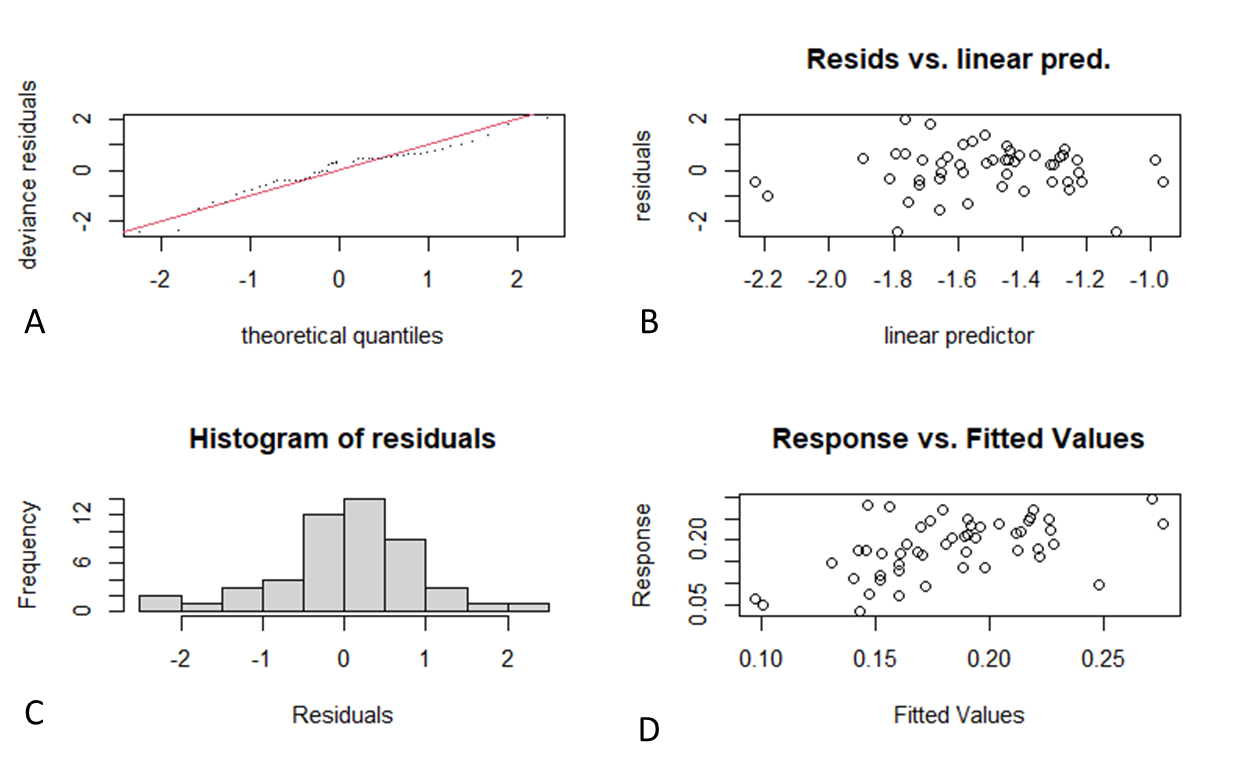


**Fig. S8**: Model’s diagnostic for the csr-strategists response variable. A Quantile plot, B residuals vs. linear predictor, C distribution of residuals, and D response vs. linear values.


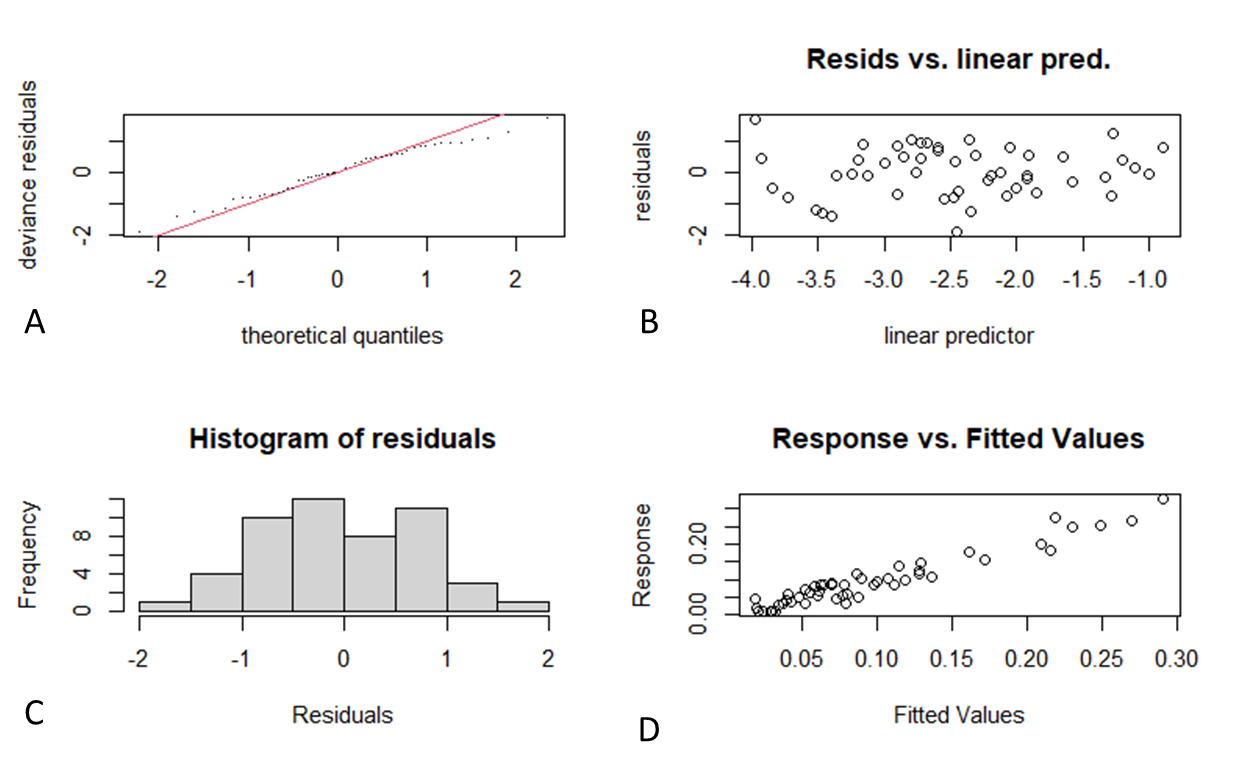


**Fig. S9**: Model’s diagnostic for the relative cover of bryophytes response variable. A Quantile plot, B residuals vs. linear predictor, C distribution of residuals, and D response vs. linear values.


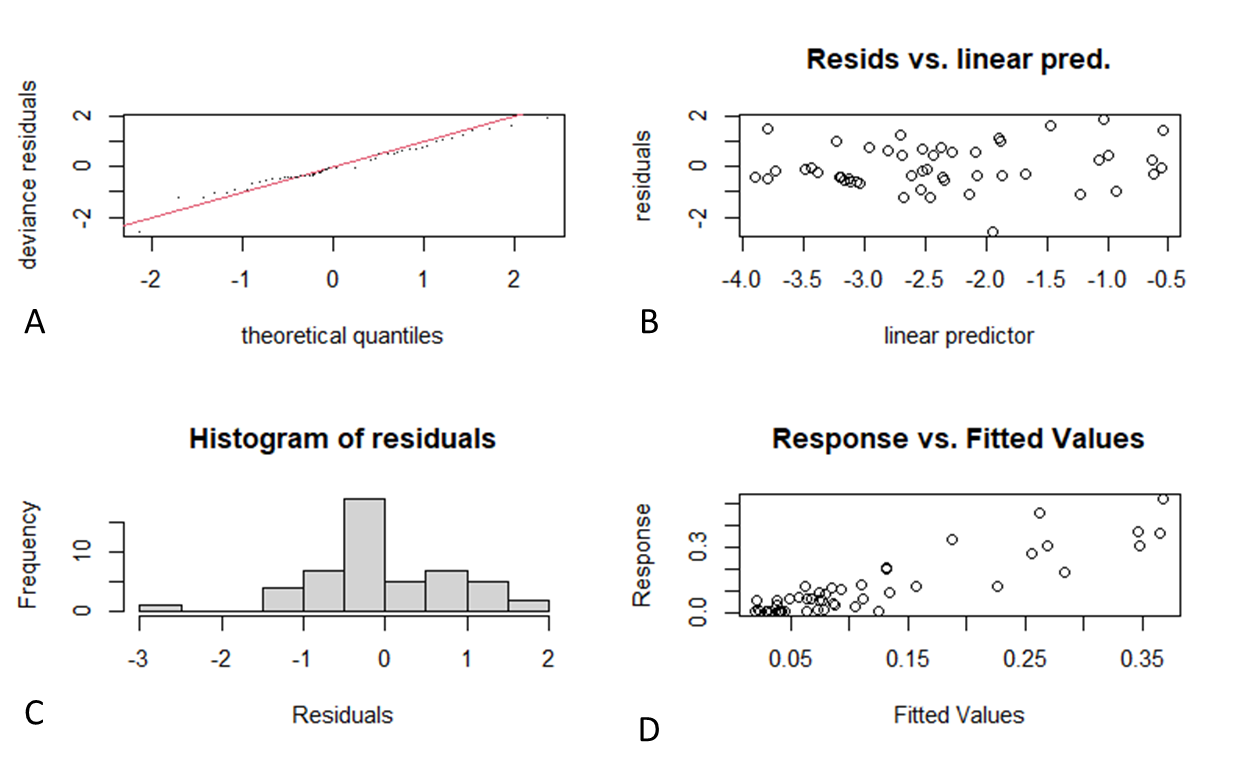


**Fig. S10**: Model’s diagnostic for the relative cover of dwarf shrubs response variable. A Quantile plot, B residuals vs. linear predictor, C distribution of residuals, and D response vs. linear values.


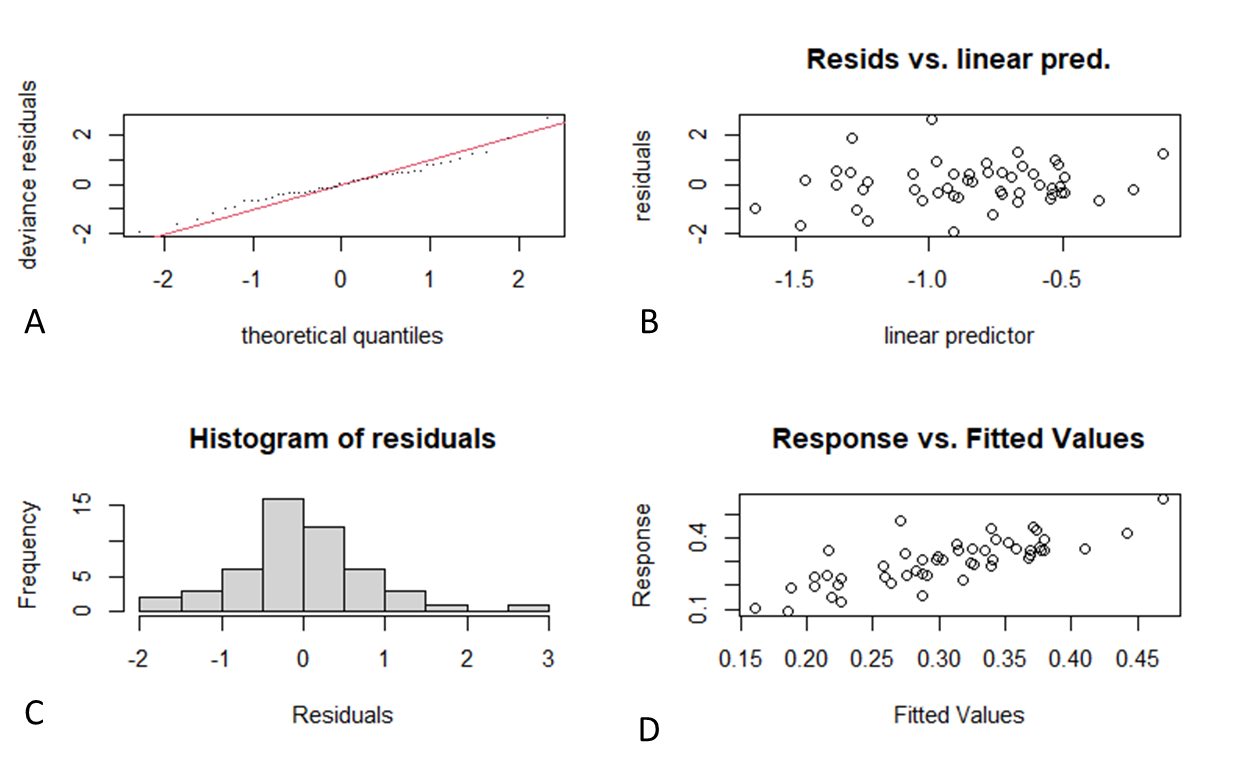


**Fig. S11**: Model’s diagnostics for the relative cover of graminoids response variable. A Quantile plot, B residuals vs. linear predictor, C distribution of residuals, and D response vs. linear values.


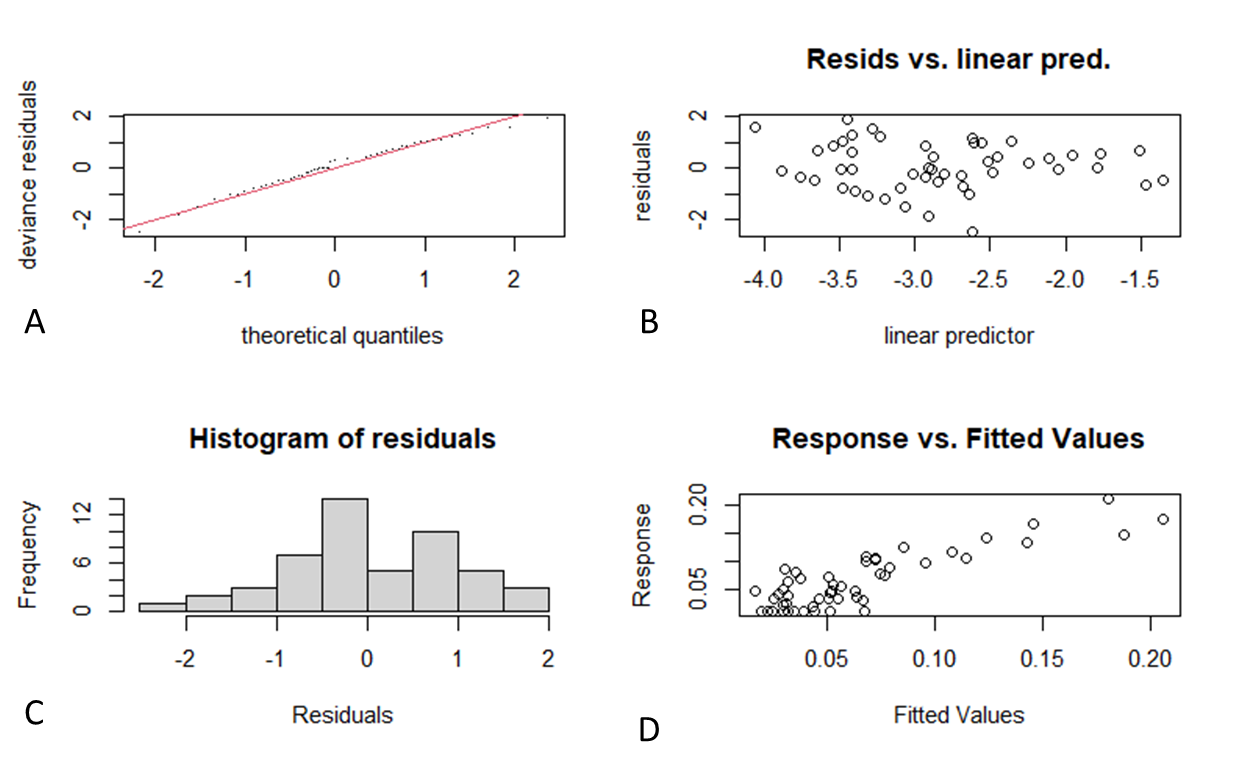


**Fig. S12**: Model’s diagnostics for the relative cover of lichens response variable. A Quantile plot, B residuals vs. linear predictor, C distribution of residuals, and D response vs. linear values.


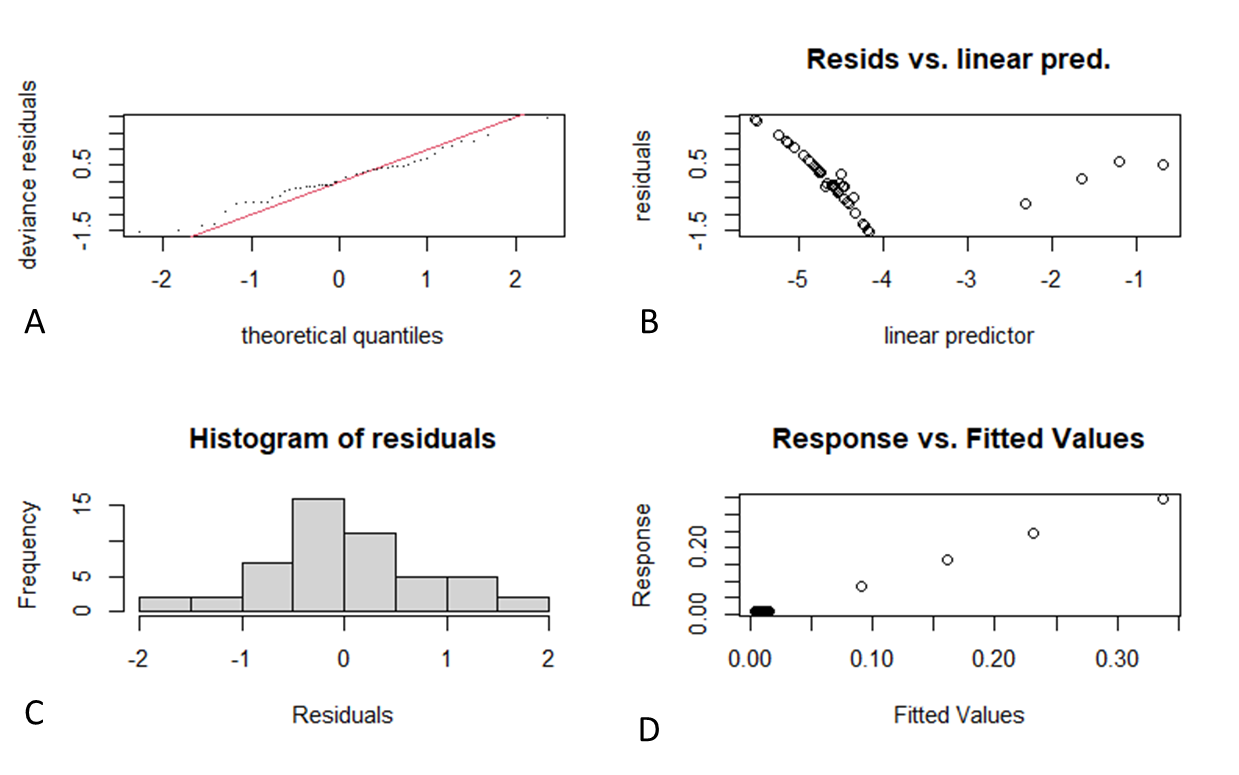


**Fig. S13**: Model’s diagnostics for the relative cover of trees response variable. A Quantile plot, B residuals vs. linear predictor, C distribution of residuals, and D response vs. linear values.


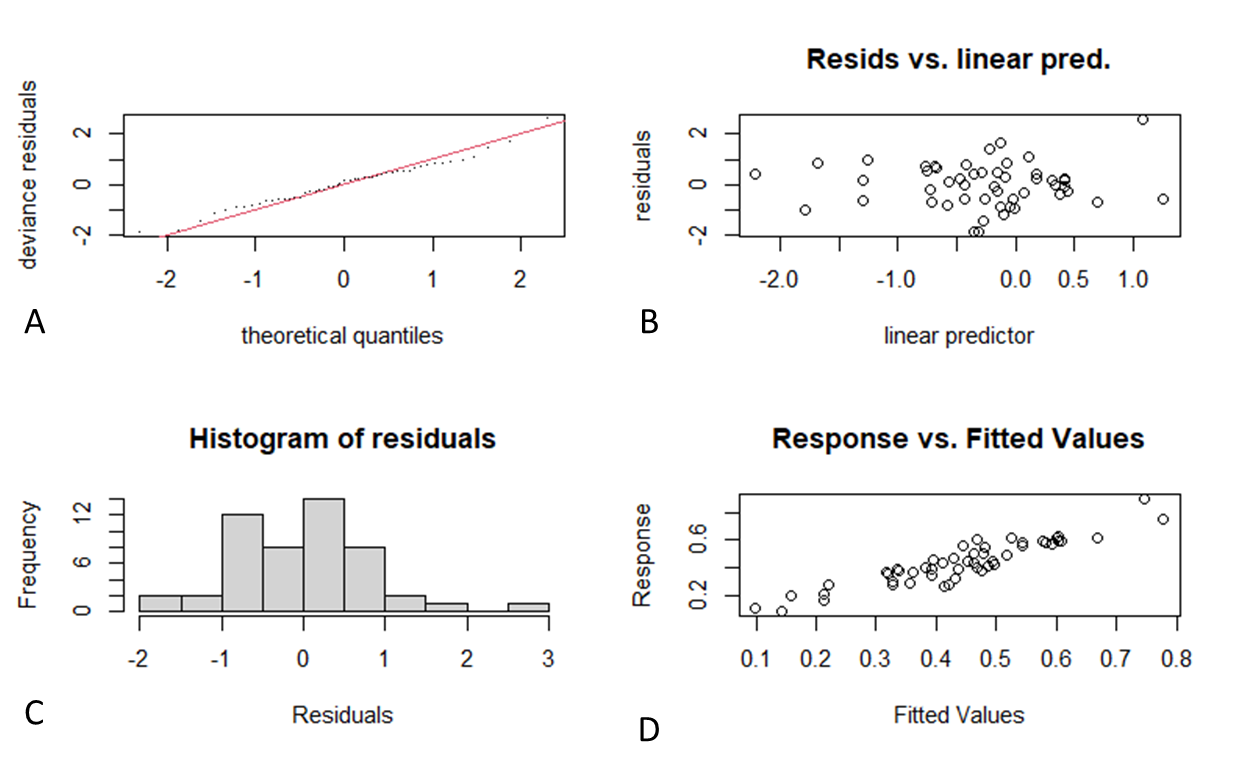


**Fig. S14**: Model’s diagnostic for the relative cover herbs response variable. A Quantile plot, B residuals vs. linear predictor, C distribution of residuals, and D response vs. linear values.


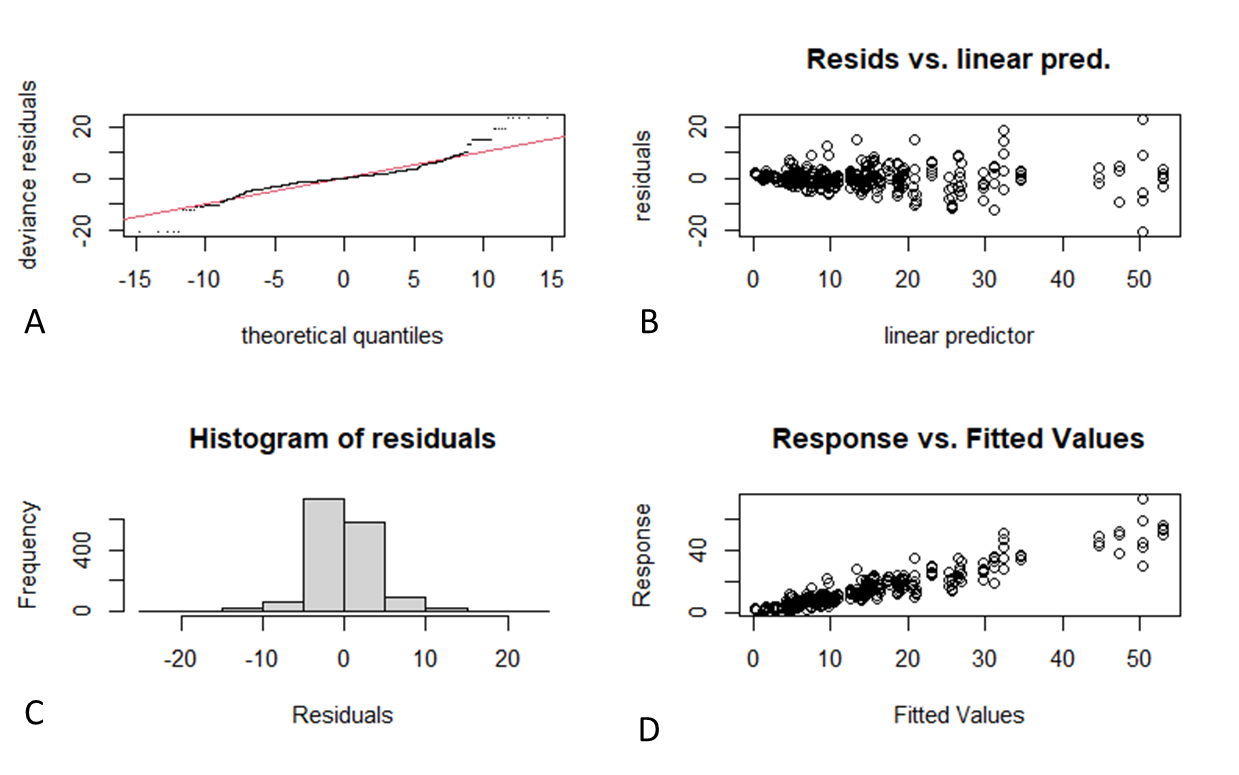


**Fig. S15**: Model’s diagnostic for the plant height response variable. A Quantile plot, B residuals vs. linear predictor, C distribution of residuals, and D response vs. linear values.


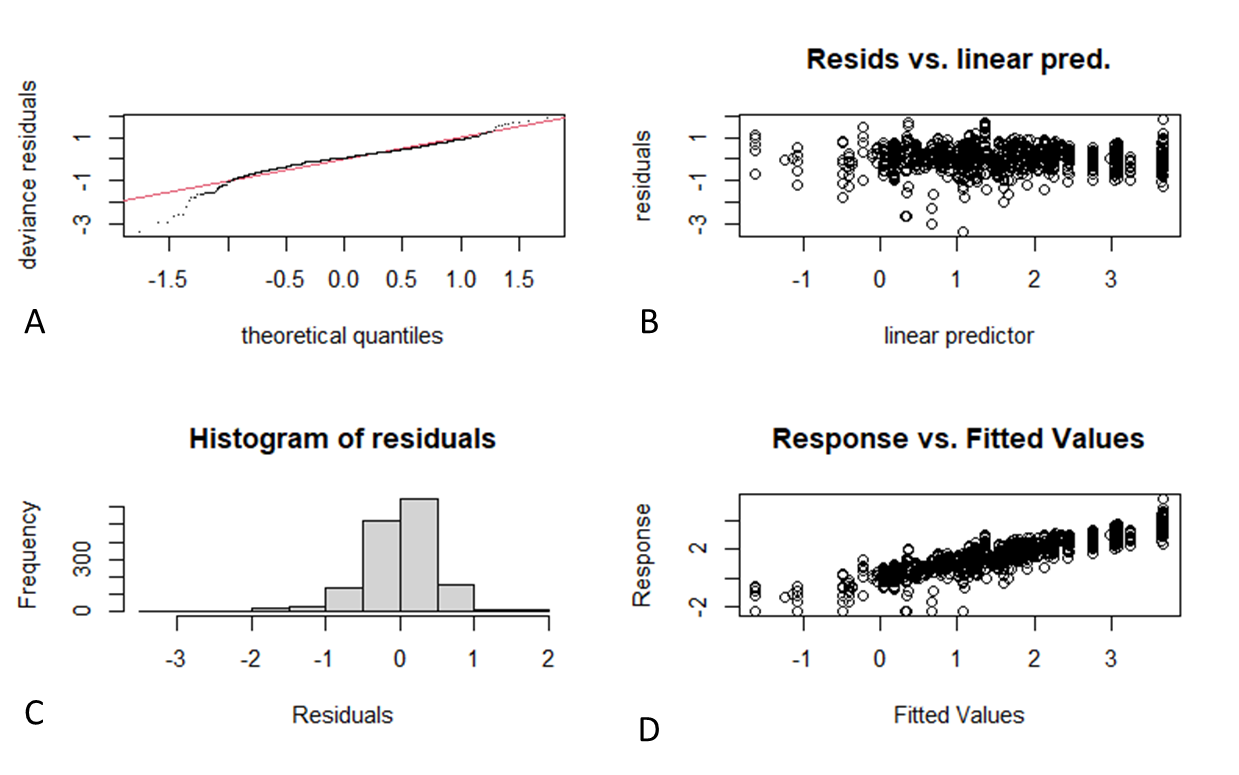


**Fig. S16**: Model’s diagnostic for the log transformed leaf dry weight response variable. A Quantile plot, B residuals vs. linear predictor, C distribution of residuals, and D response vs. linear values.


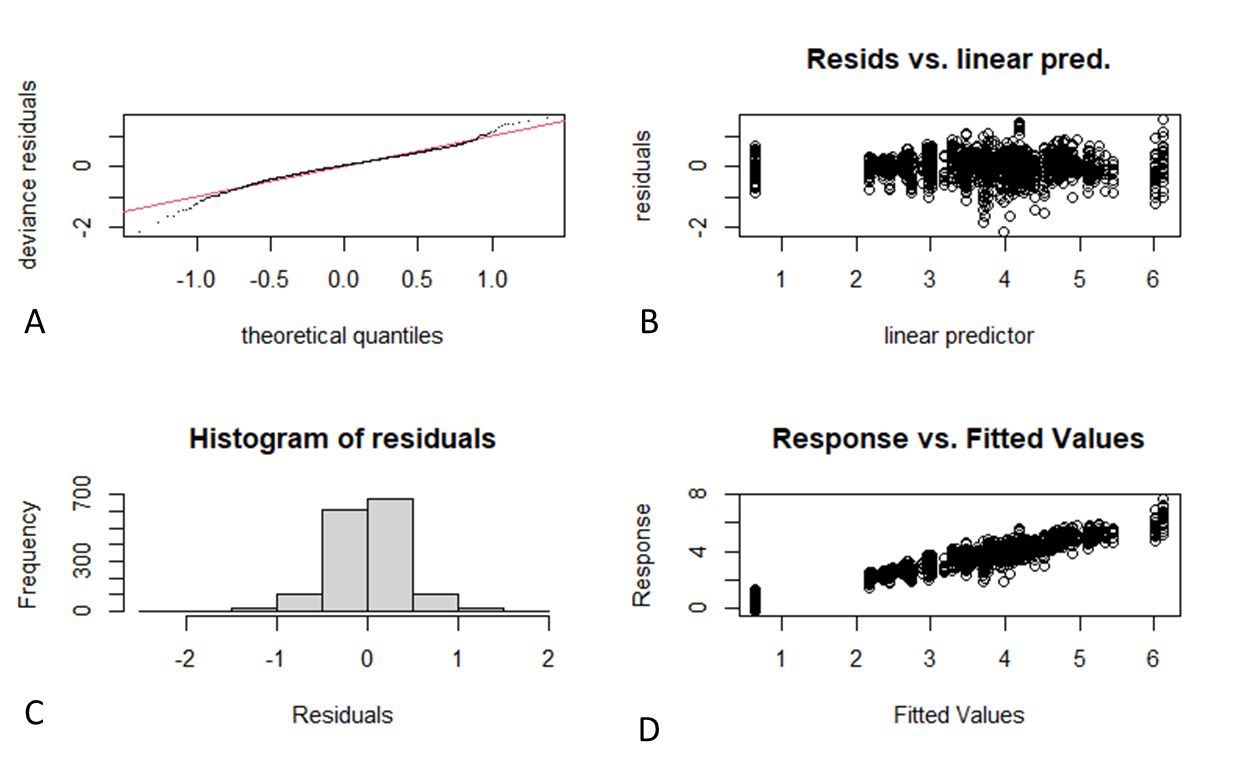


**Fig. S17**: Model’s diagnostic for the log transformed leaf area response variable. A Quantile plot, B residuals vs. linear predictor, C distribution of residuals, and D response vs. linear values.


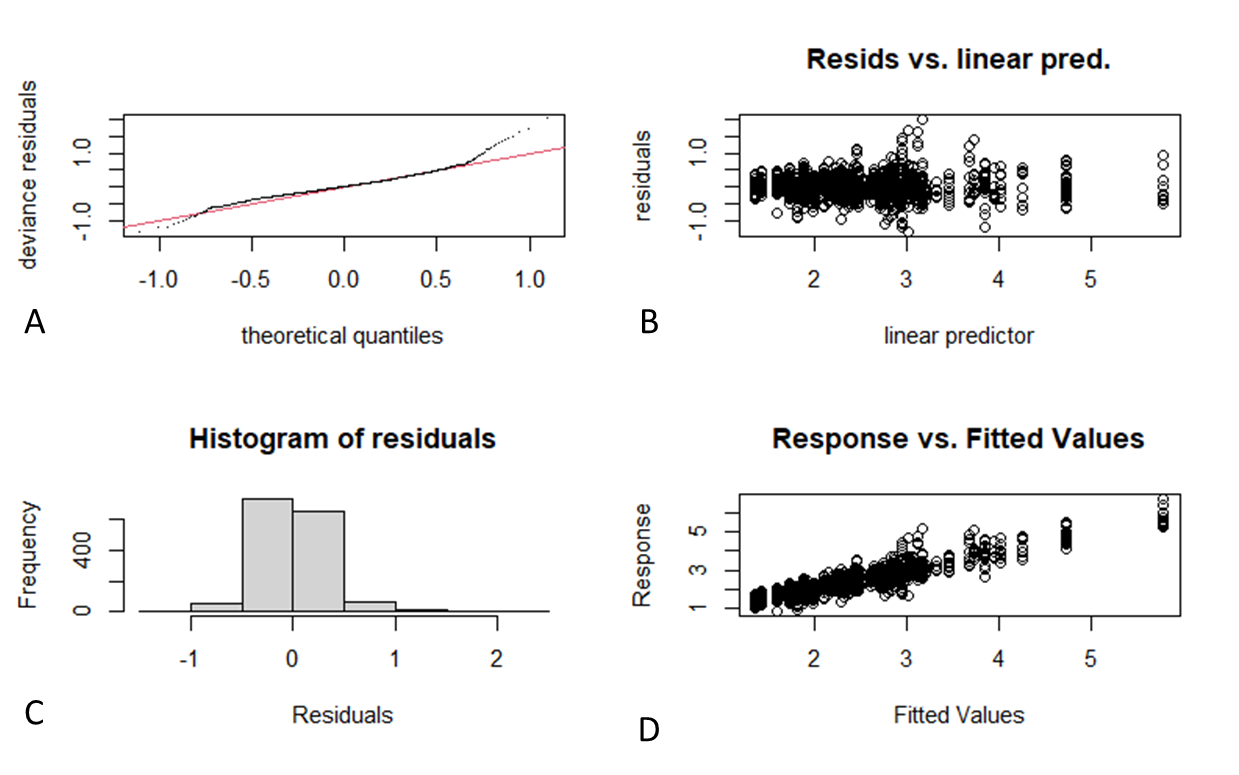


**Fig. S18**: Model’s diagnostic for the log transformed SLA response variable. A Quantile plot, B residuals vs. linear predictor, C distribution of residuals, and D response vs. linear values.

**Table S9a:** Results of generalised additive models, effects of environmental variables on total cover, species richness, relative cover of different strategy-types, life forms and temperature-types, SPI = Stream Power Index and GMD = geomorphic disturbance, with the parametric coefficients where est = estimate, SE = Standard error, dev.-expl. = deviance explained, sample size = 56; p < 0.001 ***, p < 0.01 **, p < 0.05 * and the approximate (approx.) significance (sign.) of smooth terms where estimated degrees of freedom(edf), the reference degrees of freedom (Ref. Df), the Chi-square where the significant variables are indicated by the colours.

|  | **total cover** | | | **species richness** | | | **cryophilic species** | | | **thermophilic species** | | |
| --- | --- | --- | --- | --- | --- | --- | --- | --- | --- | --- | --- | --- |
| R-sq. adj. | 0.769 |  |  | 0.606 |  |  | 0.932 |  |  | 0.930 |  |  |
| dev.-expl. (%) | 84.7 |  |  | 73.8 |  |  | 94.8 |  |  | 94.8 |  |  |
| parametric coefficients | est. | SE | p-value | est. | SE | p-value | est. | SE | p-value | est. | SE | p-value |
| climate-induced growth (RC1) | 0.858 | 0.168 | <0.001 | 0.086 | 0.038 | 0.023 | -1.277 | 0.079 | <0.001 | 1.277 | 0.079 | <0.001 |
| improved edaphic conditions (RC2) | -0.153 | 0.166 | 0.359 | -0.051 | 0.050 | 0.304 |  |  |  | -0.151 | 0.079 | 0.0562 |
| less acidic debris (RC3) |  |  |  |  |  |  | -0.026 | 0.068 | <0.7091 | 0.026 | 0.068 | 0.709 |
| GMD (disturbed) | -1.149 | 0.280 | <0.001 | 0.062 | 0.089 | 0.484 | -0.009 | 0.144 | 0.953 | -0.009 | 0.144 | 0.953 |
| SPI | -0.266 | 0.080 | <0.001 | -0.004 | 0.022 | 0.853 |  |  |  |  |  |  |
| northness | -0.379 | 0.285 | 0.183 |  |  |  | -0.101 | 0.149 | 0.486 | 0.101 | 0.149 | 0.500 |
| eastness | 0.389 | 0.228 | 0.088 |  |  |  | 0.034 | 0.097 | 0.723 | 0.034 | 0.097 | 0.723 |
| inclination | -0.021 | 0.014 | 0.143 | -0.027 | 0.005 | <0.001 | -0.009 | 0.008 | 0.260 | -0.009 | 0.008 | 0.260 |
| precipitation sum |  |  |  | <-0.01 | 0.089 | 0.735 |  |  |  | -0.001 | 0.001 | 0.093 |
|  |  |  |  |  |  |  |  |  |  |  |  |  |
| approx. sign. of smooth terms | edf | Chi.Sq.. | p-value | edf | Chi.Sq.. | p-value | edf | Chi.Sq.. | p-value | edf | Chi.Sq.. | p-value |
| s(climate-induced growth/RC1) |  |  |  |  |  |  |  |  |  |  |  |  |
| s(improved edaphic conditions/RC2) |  |  |  |  |  |  | 1.00 | 3.645 | 0.056 |  |  |  |
| s(less acidic debris/RC3) | 1.923 | 2.219 | 0.388 | 3.058 | 19.25 | <0.001 |  |  |  |  |  |  |
| s(SPI) |  |  |  |  |  |  | 1.802 | 6.079 | 0.077 | 1.802 | 6.079 | 0.077 |
| s(northness) |  |  |  | 6.663 | 26.40 | <0.001 |  |  |  |  |  |  |
| s(eastness) |  |  |  | 2.414 | 16.64 | 0.002 |  |  |  | 3.37 | 4.16 | 5.6 |
| s(inclination) |  |  |  |  |  |  |  |  |  |  |  |  |
| s(precipitation sum) | 4.394 | 17.625 | 0.007 |  |  |  | 1.000 | 2.830 | 0.093 |  |  |  |

| . | p<0.10 |  | p<0.05 |  | p<0.01 |  | p<0.001 |
| --- | --- | --- | --- | --- | --- | --- | --- |

**Table S9b:** Continuing

|  | **competitive species** | | | **ruderal species** | | | **stress-tolerant species** | | | **csr-strategists** | | |
| --- | --- | --- | --- | --- | --- | --- | --- | --- | --- | --- | --- | --- |
| R-sq. adj. | 0.742 |  |  | 0.273 |  |  | 0.606 |  |  | 0.122 |  |  |
| dev.-expl. (%) | 80.7 |  |  | 67.1 |  |  | 72.3 |  |  | 34.2 |  |  |
| parametric coefficients | est. | SE | p-value | est. | SE | p-value | est. | SE | p-value | est. | SE | p-value |
| climate-induced growth (RC1) | 0.410 | 0.078 | <0.001 |  |  |  | -0.190 | 0.069 | 0.006 | -0.161 | 0.073 | 0.028 |
| improved edaphic conditions (RC2) | -0.385 | 0.092 | <0.001 | -0.089 | 0.134 | 0.506 | 0.261 | 0.087 | 0.003 | 0.140 | 0.085 | 0.099 |
| less acidic debris (RC3) | -0.090 | 0.066 | 0.175 |  |  |  | -0.007 | 0.063 | 0.916 | 0.069 | 0.066 | 0.300 |
| GMD (disturbed) | -0.111 | 0.150 | 0.459 | 0.045 | 0.202 | 0.823 | 0.217 | 0.144 | 0.134 | -0.210 | 0.150 | 0.161 |
| SPI | 0.057 | 0.040 | 0.157 |  |  |  | -0.052 | 0.041 | 0.197 |  |  |  |
| northness | -0.108 | 0.181 | 0.551 | -0.177 | 0.207 | 0.391 | -0.059 | 0.163 | 0.718 | 0.171 | 0.136 | 0.207 |
| eastness |  |  |  | -0.168 | 0.134 | 0.208 |  |  |  |  |  |  |
| inclination | -0.029 | 0.008 | <0.001 |  |  |  | 0.028 | 0.008 | <0.001 | 0.001 | 0.008 | 0.924 |
| precipitation sum |  |  |  | -0.003 | 0.001 | 0.002 | 0.001 | 0.001 | 0.451 | <-0.001 | 0.001 | 0.8867 |
|  |  |  |  |  |  |  |  |  |  |  |  |  |
| approx. sign. of smooth terms | edf | Chi.Sq. | p-value | edf | Chi.Sq. | p-value | edf | Chi.Sq. | p-value | edf | Chi.Sq.. | p-value |
| s(climate-induced growth/RC1) |  |  |  | 3.642 | 19.890 | <0.001 |  |  |  |  |  |  |
| s(improved edaphic conditions/RC2) |  |  |  |  |  |  |  |  |  |  |  |  |
| s(less acidic debris/RC3) |  |  |  | 4.192 | 19.462 | 0.002 |  |  |  |  |  |  |
| s(SPI) |  |  |  | 1.937 | 4.547 | 0.128 |  |  |  | 1.410 | 0.66 | 0.562 |
| s(northness) |  |  |  |  |  |  |  |  |  |  |  |  |
| s(eastness) | 4.063 | 12.280 | 0.033 |  |  |  | 4.914 | 15.02 | 0.019 | 1.000 | 4.29 | 0.038 |
| s(inclination) |  |  |  | 1.558 | 1.971 | 0.427 |  |  |  |  |  |  |
| s(precipitation sum) | 2.243 | 4.889 | 0.144 |  |  |  |  |  |  |  |  |  |

| . | p<0.10 |  | p<0.05 |  | p<0.01 |  | p<0.001 |
| --- | --- | --- | --- | --- | --- | --- | --- |

**Table S9c:** Continuing

|  | **bryophytes** | | | **dwarf shrubs** | | | **graminoids** | | |
| --- | --- | --- | --- | --- | --- | --- | --- | --- | --- |
| R-sq. adj. | 0.868 |  |  | 0.682 |  |  | 0.432 |  |  |
| dev.-expl. (%) | 89.8 |  |  | 77.4 |  |  | 62.7 |  |  |
| parametric coefficients | est. | SE | p-value | est. | SE | p-value | est. | SE | p-value |
| climate-induced growth (RC1) |  |  |  | 0.391 | 0.146 | 0.007 |  |  |  |
| improved edaphic conditions (RC2) | -0.215 | 0.110 | 0.051 | -0.819 | 0.169 | <0.001 |  |  |  |
| less acidic debris (RC3) | -0.306 | 0.074 | <0.001 |  |  |  | 0.042 | 0.060 | 0.488 |
| GMD (disturbed) | 0.306 | 0.185 | 0.098 | -0.052 | 0.300 | 0.861 |  |  |  |
| SPI |  |  |  | 0.208 | 0.072 | 0.004 |  |  |  |
| northness |  |  |  |  |  |  | -0.004 | 0.156 | 0.974 |
| eastness |  |  |  |  |  |  | -0.147 | 0.091 | 0.107 |
| inclination | -0.029 | 0.012 | 0.017 | -0.05 | 0.02 | 0.004 | 0.001 | 0.007 | 0.819 |
| precipitation sum | <0.001 | <0.001 | 0.903 |  |  |  | 0.001 | 0.001 | 0.153 |
|  |  |  |  |  |  |  |  |  |  |
| approx. sign. of smooth terms | edf | Chi.Sq.. | p-value | edf | Chi.Sq.. | p-value | edf | Chi.Sq.. | p-value |
| s(climate-induced growth/RC1) | 5.538 | 38.73 | <0.001 |  |  |  | 2.883 | 10.25 | 0.041 |
| s(improved edaphic conditions/RC2) |  |  |  |  |  |  | 2.392 | 9.59 | 0.028 |
| s(less acidic debris/RC3) |  |  |  | 2.649 | 8.23 | 0.047 |  |  |  |
| s(SPI) | 2.259 | 10.84 | 0.009 |  |  |  | 3.500 | 8.66 | 0.075 |
| s(northness) | 1.001 | 27.62 | <0.001 | 2.90 | 4.66 | 0.319 |  |  |  |
| s(eastness) | 5.754 | 27.36 | <0.001 | 3.798 | 20.98 | <0.002 |  |  |  |
| s(inclination) |  |  |  |  |  |  |  |  |  |
| s(precipitation sum) |  |  |  | 2.005 | 3.49 | 0.246 |  |  |  |

| . | p<0.10 |  | p<0.05 |  | p<0.01 |  | p<0.001 |
| --- | --- | --- | --- | --- | --- | --- | --- |

**Table S9d:** Continuing

|  | **lichens** | | | **trees** | | | **herbs** | | |
| --- | --- | --- | --- | --- | --- | --- | --- | --- | --- |
| R-sq. adj. | 0.670 |  |  | 0.995 |  |  | 0794 |  |  |
| dev.-expl. (%) | 63.3 |  |  | 98.2 |  |  | 85.7 |  |  |
| parametric coefficients | est. | SE | p-value | est. | SE | p-value | est. | SE | p-value |
| climate-induced growth (RC1) | -0.276 | 0.097 | 0.005 | 0.362 | 0.089 | <0.001 | -0.258 | 0.066 | <0.001 |
| improved edaphic conditions (RC2) | -0.035 | 0.107 | 0.743 | -0.236 | 0.097 | 0.015 | 0.589 | 0.075 | <0.001 |
| less acidic debris (RC3) | -0.300 | 0.073 | <0.001 | -0.097 | 0.074 | 0.188 | 0.249 | 0.064 | <0.001 |
| GMD (disturbed) | -0.569 | 0.198 | 0.004 | -0.359 | 0.168 | 0.033 | 0.134 | 0.127 | 0.292 |
| SPI | 0.018 | 0.058 | 0.761 | 0.146 | 0.049 | 0.003 |  |  |  |
| northness | 0.543 | 0.169 | 0.001 |  |  |  |  |  |  |
| eastness | -0.079 | 0.112 | 0.479 |  |  |  |  |  |  |
| inclination | -0.010 | 0.010 | 0.305 | -0.037 | 0.012 | 0.001 | 0.034 | 0.001 | <0.001 |
| precipitation sum | -0.001 | 0.001 | 0.172 |  |  |  | -0.001 | 0.001 | 0.265 |
|  |  |  |  |  |  |  |  |  |  |
| approx. sign. of smooth terms | edf | Chi.Sq.. | p-value | edf | Chi.Sq.. | p-value | edf | Chi.Sq.. | p-value |
| s(climate-induced growth/RC1) |  |  |  |  |  |  |  |  |  |
| s(improved edaphic conditions/RC2) |  |  |  |  |  |  |  |  |  |
| s(less acidic debris/RC3) |  |  |  |  |  |  |  |  |  |
| s(SPI) |  |  |  |  |  |  | 2.000 | 23.79 | <0.001 |
| s(northness) |  |  |  | 5.969 | 23.04 | 0.011 | 4.127 | 22.00 | <0.001 |
| s(eastness) |  |  |  | 3.677 | 45.48 | <0.001 | 1.000 | 0.09 | 0.766 |
| s(inclination) |  |  |  |  |  |  |  |  |  |
| s(precipitation sum) |  |  |  | 8.135 | 102.68 | <0.001 |  |  |  |

| . | p<0.10 |  | p<0.05 |  | p<0.01 |  | p<0.001 |
| --- | --- | --- | --- | --- | --- | --- | --- |

**Table S10:** Results of generalised additive models, effects of environmental variables on plant height, leaf dry weight, leaf area, and SLA (specific leaf area), SPI = Stream Power Index and GMD = geomorphic disturbance, with the parametric coefficients where est = estimate, SE = Standard error, dev.-expl. = deviance explained, sample size = 56; p < 0.001 ***, p < 0.01 **, p < 0.05 * and the approximate (approx.) significance (sign.) of smooth terms where estimated degrees of freedom(edf), the reference degrees of freedom (Ref. Df), the Chi-square where the significant variables are indicated by the colours.

|  | **plant height (cm)** | | | **dry weight (mg)** | | | **leaf area (mm²)** | | | **SLA (mm²mg^-1^)** | | |
| --- | --- | --- | --- | --- | --- | --- | --- | --- | --- | --- | --- | --- |
| R-sq. adj. | 0.868 |  |  | 0.817 |  |  | 0.874 |  |  | 0.814 |  |  |
| dev.-expl. (%) | 87.3 |  |  | 82.4 |  |  | 87.8 |  |  | 82.0 |  |  |
| parametric coefficients | est. | SE | p-value | est. | SE | p-value | est. | SE | p-value | est. | SE | p-value |
| climate-induced growth (RC1) |  |  |  | 0.200 | 0.099 | 0.044 | 0.560 | 0.135 | <0.001 | 0.313 | 0.093 | <0.001 |
| improved edaphic conditions (RC2) | -0.299 | 0.711 | 0.720. | 0.142 | 0.060 | 0.017 | 0.004 | 0.043 | 0.928 | -0.137 | 0.031 | <0.001 |
| less acidic debris (RC3) |  |  |  | -0.112 | 0.072 | 0.0118 | 0.273 | 0.065 | <0.001 |  |  |  |
| GMD (disturbed) | -0.479 | 1.319 | 0.665 | 0.043 | 0.0786 | 0.557 | -0.160 | 0.074 | 0.030 | 0.243 | 0.076 | 0.001 |
| SPI | -0.399 | 0.359 | 0.255 |  |  |  | -0.068 | 0.041 | 0.093 | -0.127 | 0.027 | <0.001 |
| northness | 7.230 | 1.929 | <0.001 | -0.022 | 0.146 | 0.883 |  |  |  | -0.112 | 0.129 | 0.385 |
| eastness | -4.366 | 1.661 | 0.010 | -0.628 | 0.146 | <0.001 | 0.049 | 0.167 | 0.771 |  |  |  |
| inclination | 0.556 | 0.091 | <0.000 |  |  |  |  |  |  |  |  |  |
| precipitation sum |  |  |  |  |  |  |  |  |  |  |  |  |
| graminoids | -3.003 | 6.076 | 0.638 | -1.502 | 0.568 | 0.008 | -0.854 | 0.585 | 0.145 | 1.069 | 0.344 | 0.002 |
| herb | -13.004 | 5.454 | 0.015 | -1.204 | 0.5123 | 0.019 | -0.506 | 0.528 | 0.340. | 1.235 | 0.309 | <0.000 |
|  |  |  |  |  |  |  |  |  |  |  |  |  |
| approx. sign. of smooth terms | edf | Ref.Df. | p-value | edf | Ref.Df. | Chi.Sq. | edf | Ref.Df. | p-value | edf | Ref.Df. | p-value |
| s(climate-induced growth/RC1) | 7.858 | 8.071 | <0.001 |  |  |  |  |  |  |  |  |  |
| s(improved edaphic conditions/RC2) |  |  |  |  |  |  |  |  |  |  |  |  |
| s(less acidic debris/RC3) | 7.767 | 8.099 | <0.001 |  |  |  |  |  |  | 2.259 | 2.490 | 0.009 |
| s(SPI) |  |  |  | 7.484 | 7.997 | <0.001 |  |  |  |  |  |  |
| s(northness) |  |  |  |  |  |  | 5.978 | 6.477 | <0.001 |  |  |  |
| s(eastness) |  |  |  |  |  |  |  |  |  | 7.697 | 8.197 | <0.001 |
| s(inclination) |  |  |  | 6.282 | 6.900 | <0.001 | 7.366 | 7.929 | <0.001 | 5.971 | 6.543 | <0.001 |
| s(precipitation sum) | 8.365 | 8.563 | <0.001 | 6.198 | 6.744 | <0.001 | 8.145 | 8.399 | <0.001 | 6.928 | 7.321 | <0.001 |
| s(species) | 24.034 | 25.000 | <0.001 | 24.427 | 25.000 | <0.001 | 24.565 | 25.000 | <0.001 | 25.241 | 26.000 | <0.001 |

| . | p<0.10 |  | p<0.05 |  | p<0.01 |  | p<0.001 |
| --- | --- | --- | --- | --- | --- | --- | --- |

**
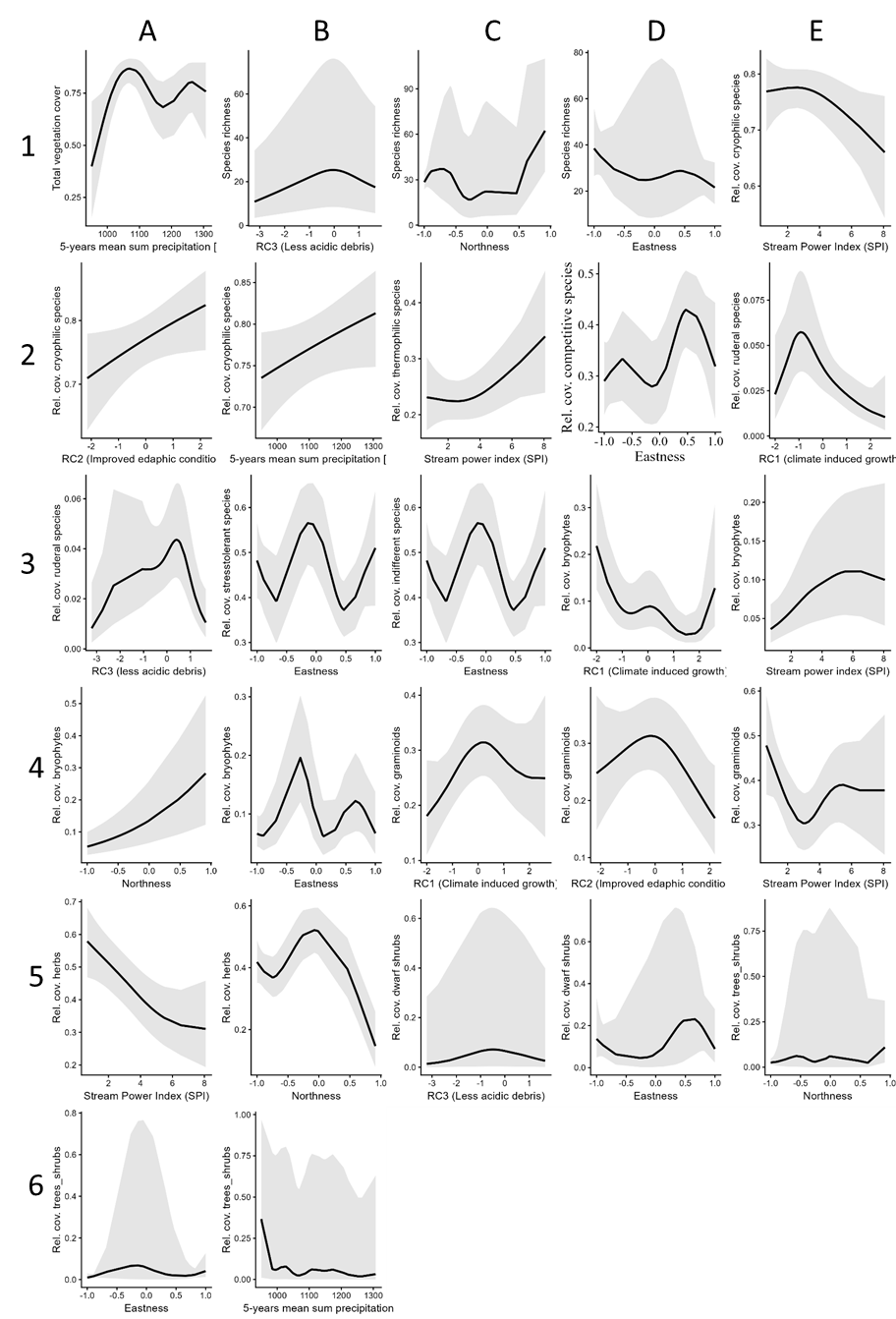
**

**Fig. S19:** Significant results of the smooth terms of the generalised additive models;1A – total cover, 1B-D – species richness, 1 E and 2A/B – relative cover cryophilic species, 2C – relative cover thermophilic species, 2D – relative cover competitive species, 2E and 3A – relative cover ruderal species, 3B – relative cover stresstolerant species, 3C – relative cover indifferent species, 3D/E and 4A/B – relative cover bryophytes, 4C-E relative cover graminoids, 5A/B – relative cover herbs, 5C/D- relative cover dwarf shrubs, 5 E and 6A/B- relative cover trees.


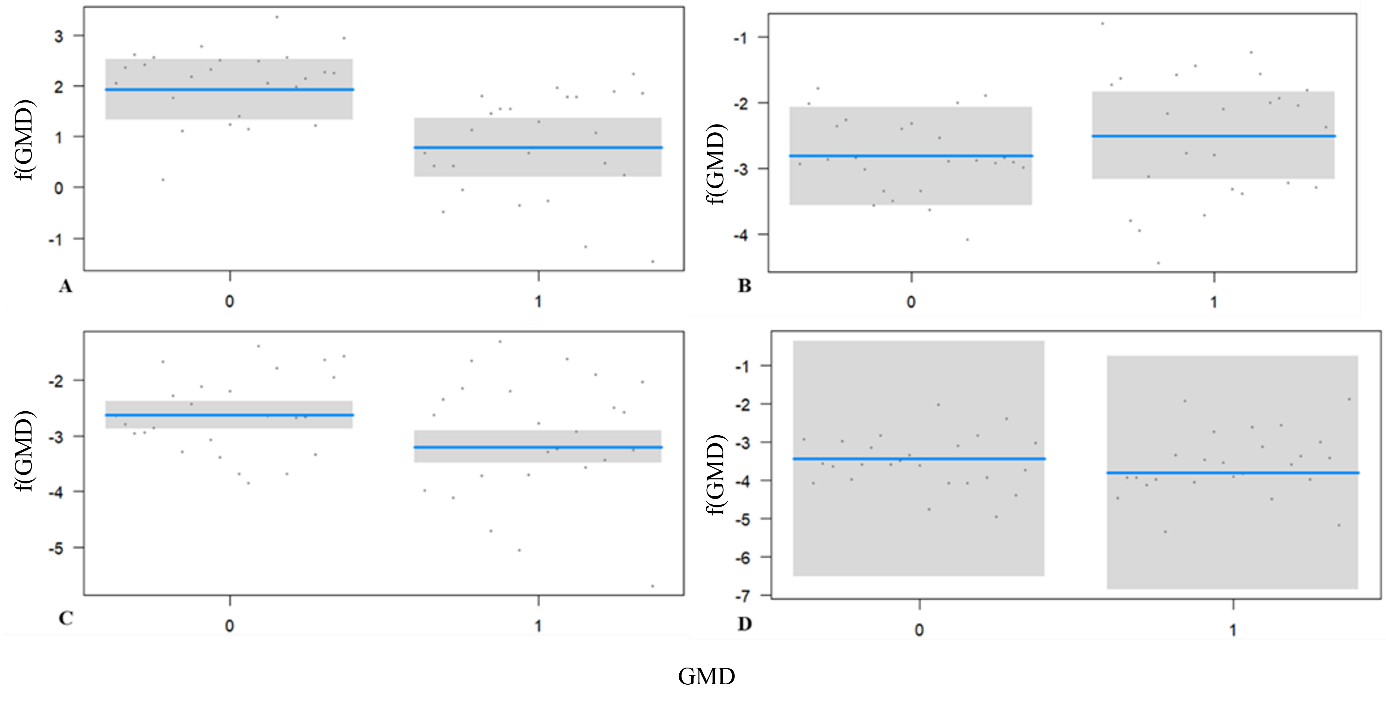


**Fig. S20:** Significant results categorical variable GMD (geomorphic disturbance; 0 = undisturbed, 1 = disturbed) for A total cover, B relative cover of bryophytes, C relative cover of lichens, and D relative cover of trees.


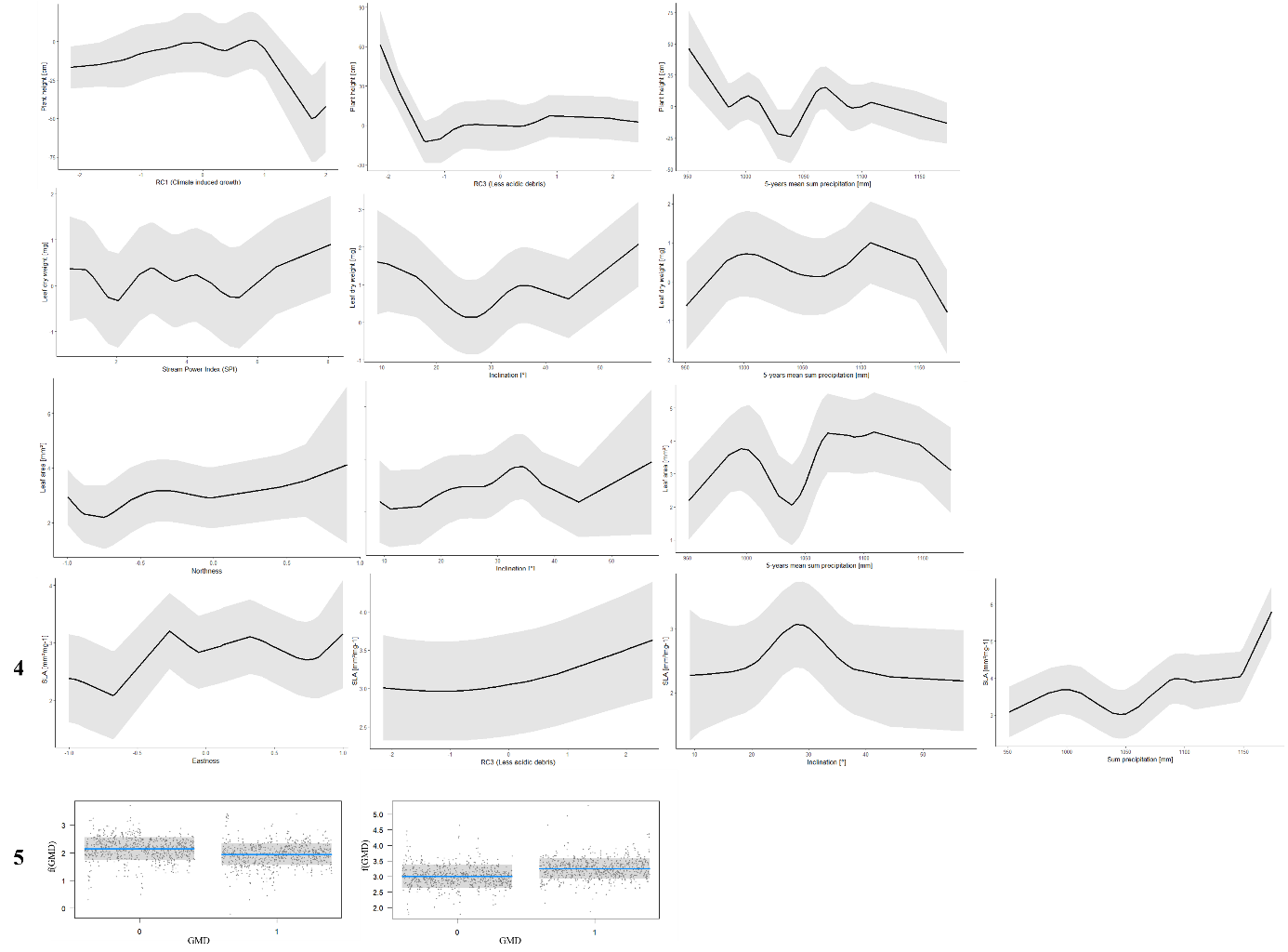


**Fig. S21:** Significant results of the smooth terms of the generalised additive models with the traits in the rows (1 plant height, 2 leaf dry weight, 3 leaf area, and 4 SLA) and the response variables in the columns as well as in row 5 the significant difference in undisturbed (0) and disturbed (1) plots (GMD = geomorphic disturbance) for the leaf area and SLA.

**
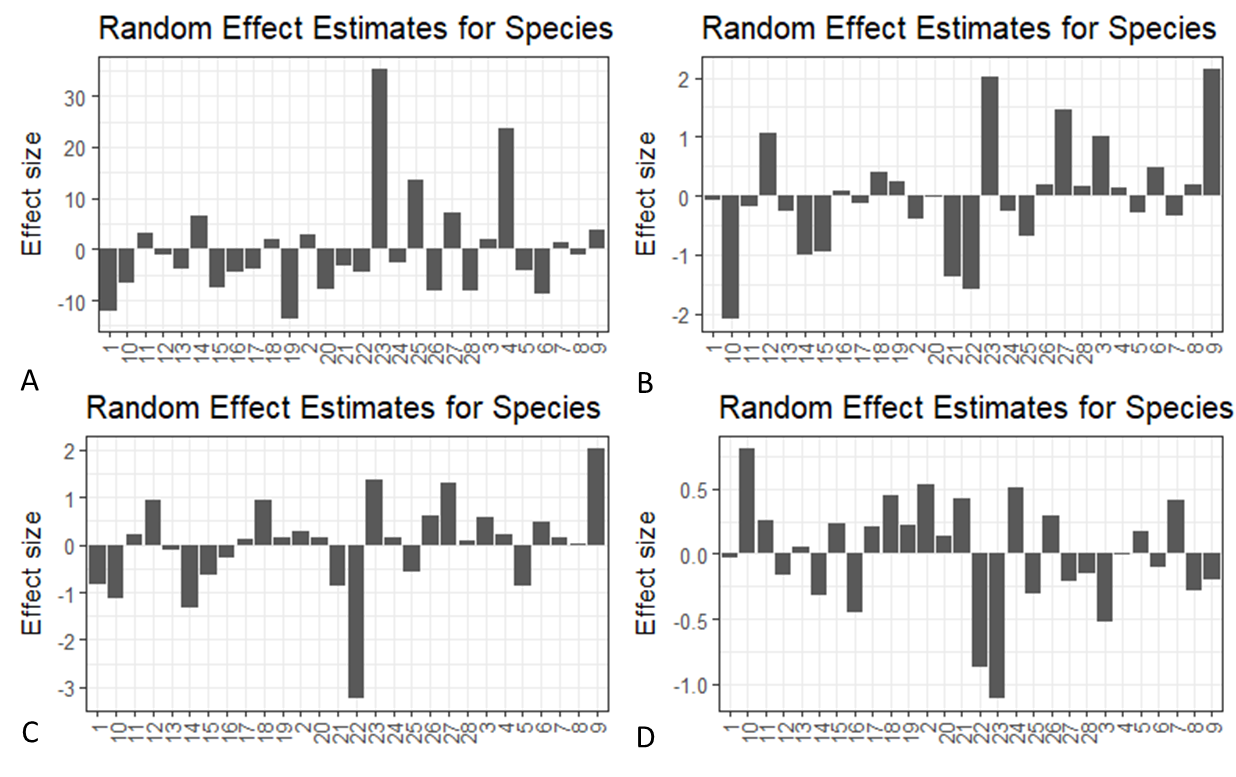
**

**Fig. S22.** Random effect estimates for the species for A plant height, B leaf dry weight, C leaf area, and D SLA whereas the numbers indicate the different species given in Table S8.

**Table S11:** Means of the measured traits of species grown on undisturbed (0) and disturbed (1) sites. M = mean, SD = standard deviation, n = number of measurements, p-value of the Kruskal-Wallis-test, the different colour intensities indicate the different significant levels.

grasses, herbs incl. legumes, dwarf shrubs

| **Species** | | **plant height (cm)** | **dry weight (mg)** | **leaf area (mm²)** | **SLA (mm²mg^-1^)** |
| --- | --- | --- | --- | --- | --- |
| *Agrostis rupestris* | *n* | 10 | 50 | 50 | 50 |
|  | *0* | 7.0±1.1 | 0.9±0.2 | 14.2±2.8 | 16.0±3.5 |
|  | *1* | 7.2±0.9 | 1.2±0.3 | 15.6±3.6 | 14.3±4.8 |
|  | *p* | n.s. | * | n.s. | n.s. |
| *Anthoxanthum odoratum* agg. | *n* | 23 | 77 | 77 | 77 |
|  | *0* | 29.6±15.3 | 1.4±1.1 | 66.5.9±53.3 | 111.7±173.5 |
|  | *1* | 23.2±7.0 | 7.5±6.2 | 114.8±85.6 | 25.9±29.6 |
|  | *p* | . | *** | ** | *** |
| *Avenella flexuosa* | *n* | 26 | 95 | 95 | 95 |
|  | *0* | 51.5±4.2 | 4.5±1.8 | 45.9±13.3 | 10.7±2.5 |
|  | *1* | 49.7±14.0 | 5.1±2.8 | 50.4±25.4 | 10.9±2.5 |
|  | *p* | n.s. | n.s. | n.s. | n.s. |
| *Carex curvula* | *n* | 45 | 159 | 136 | 136 |
|  | *0* | 12.0±3.7 | 8.4±4.6 | 110.5±65.2 | 12.8±3.3 |
|  | *1* | 7.6±5.1 | 4.8±3.9 | 57.9±47.9 | 15.6±15.4 |
|  | *p* | *** | *** | *** | n.s. |
| *Festuca halleri* | *n* | 10 | 50 | 50 | 50 |
|  | *0* | 13.4±1.6 | 2.08±0.6 | 18.3±6.8 | 9.0±2.6 |
|  | *1* | 12.5±2.1 | 3.4±0.6 | 23.5±5.5 | 7.1±1.7 |
|  | *p* | ** | *** | ** | ** |
| *Helictochloa versicolor* | *n* | 27 | 67 | 54 | 54 |
|  | *0* | 23.5±5.1 | 7.5±3.4 | 152.8±54.2 | 21.5±5.5 |
|  | *1* | 28.6±5.1 | 4.7±2.8 | 97.1±51.60 | 21.9±5.7 |
|  | *p* | * | * | *. | n.s. |
| *Juncus trifidus* | *n* | 44 | 155 | 109 | 109 |
|  | *0* | 18.0±2.9 | 3.2±2.0 | 73.3±57.5 | 38.7±58.2 |
|  | *1* | 22.4±5.7 | 3.4±1.9 | 44.5±24.5 | 18.0±24.4 |
|  | *p* | *** | n.s. | * | ** |
| *Oreochloa disticha* | *n* | 6 | 18 | 18 | 18 |
|  | *0* | 19.7±2.6 | 7.7±1.5 | 79.9±14.9 | 10.4±1.0 |
|  | *1* | 16.4±5.9 | 6.6±2.6 | 71.3±24.5 | 11.0±1.3 |
|  | *p* | n.s. | n.s. | n.s. | n.s. |
| *Cardamine resedifolia* | *n* | 10 | 50 | 50 | 50 |
|  | *0* | 6.8±1.4 | 1.2±0.1 | 25.0±7.8 | 21.0±6.4 |
|  | *1* | 3.3±0.7 | 1.0±0.1 | 17.9±5.5 | 17.3±5.1 |
|  | *p* | *** | *** | ** | n.s. |
| *Geum montanum* | *n* | 16 | 68 | 68 | 68 |
|  | *0* | 20.2±3.4 | 67.2±48.9 | 704.4±453.6 | 11.0±1.7 |
|  | *1* | 14.0±1.9 | 38.8±19.4 | 1070.5±955.34 | 25.9±22.0 |
|  | *p* | *** | ** | n.s. | *** |
| *Gnaphalium supinum* | *n* | 6 | 17 | 18 | 17 |
|  | *0* | 1.0±0.3 | 0.3±0.2 | 15.0±6.2 | 79.1±34.9 |
|  | *1* | 1.4±0.2 | 0.3±0.2 | 17.5±3.5 | 60.8±23.7 |
|  | *p* | ** | n.s. | n.s. | n.s. |
| *Jacobaea incana* | *n* | 6 | 18 | 18 | 18 |
|  | *0* | 11.1±1.0 | 14.9±3.7 | 244.2±63.3 | 16.5±2.5 |
|  | *1* | 6.2±0.5 | 11.5±3.9 | 176.0±57.1 | 15.5±1.4 |
|  | *p* | *** | . | * | n.s. |
| *Leucanthemopsis alpina* | *n* | 39 | 142 | 104 | 104 |
|  | *0* | 4.6±3.7 | 1.9±1.6 | 40.0±17.1 | 26.1±18.3 |
|  | *1* | 3.9±2.3 | 2.7±1.2 | 51.5±24.5 | 20.2±6.6 |
|  | *p* | n.s. | *** | ** | * |
| *Phyteuma hemisphaericum* | *n* | 6 | 18 | 18 | 18 |
|  | *0* | 6.7±0.5 | 2.9±0.9 | 68.1±15.2 | 24.2±3.9 |
|  | *1* | 6.0±1.6 | 3.7±0.8 | 78.6±13.7 | 21.7±3.3 |
|  | *p* | n.s. | . | n.s. | n.s. |
| *Primula glutinosa* | *n* | 10 | 50 | 50 | 50 |
|  | *0* | 5.2±0.5 | 4.2±1.3 | 72.7±20.2 | 18.0±4.9 |
|  | *1* | 6.6±0.6 | 5.0±2.2 | 75.2±19.3 | 16.3±4.4 |
|  | *p* | *** | n.s. | n.s. | n.s. |
| *Saxifraga bryoides* | *n* | 10 | 50 | 50 | 50 |
|  | *0* | 4.6±0.6 | 0.32±0.0 | 1.7±0.5 | 5.3±1.7 |
|  | *1* | 4.9±0.1 | 0.28±0.0 | 2.3±0.8 | 8.4±2.7 |
|  | *p* | n.s. | *** | ** | *** |
| *Saxifraga paniculata* | *n* | 10 | 50 | 50 | 50 |
|  | *0* | 35.6±1.5 | 13.3±4.4 | 125.3±32.9 | 10.0±2.4 |
|  | *1* | 30.3±6.3 | 12.0±4.8 | 69.5±18.6 | 6.3±1.6 |
|  | *p* | *** | n.s. | *** | *** |
| *Silene rupestris* | *n* | 11 | 43 | 43 | 43 |
|  | *0* | 7.4±1.2 | 2.7±0.7 | 42.6±8.3 | 15.9±2.5 |
|  | *1* | 8.7±2.0 | 1.7±0.9 | 123.6±56.4 | 101.1±68.7 |
|  | *p* | n.s | * | *** | *** |
| *Trifolium alpinum* | *n* | 16 | 52 | 52 | 52 |
|  | *0* | 4.6±0.6 | 8.6±2.9 | 187.5.0±57.9 | 22.6±4.4 |
|  | *1* | 5.8±2.1 | 11.3±4.6 | 236.9±75.4 | 21.7±2.9 |
|  | *p* | n.s. | n.s. | n.s. | n.s. |
| *Veronica bellidioides* | *n* | 10 | 50 | 50 | 50 |
|  | *0* | 5.5±4.5 | 7.2±2.5 | 71.0±14.5 | 10.7±3.0 |
|  | *1* | 5.9±4.4 | 5.3±2.9 | 57.8±18.8 | 15.0±9.4 |
|  | *p* | . | * | ** | n.s. |
| *Arctostaphylos* *uva-ursi* | *n* | 10 | 50 | 50 | 50 |
|  | *0* | 11.0±5.7 | 17.1±6.5 | 72.8±17.4 | 4.6±1.0 |
|  | *1* | 8.9±1.1 | 22.5±8.7 | 77.4±16.7 | 3.7±0.9 |
|  | *p* | n.s. | * | n.s. | ** |
| *Daphne striata* | *n* | 30 | 149 | 100 | 100 |
|  | *0* | 10.8±4.3 | 4.4±0.8 | 47.3±15.4 | 10.8±3.0 |
|  | *1* | 10.1±3.3 | 4.0±0.5 | 44.9±10.5 | 11.3±2.4 |
|  | *p* | n.s. | ** | n.s. | n.s. |
| *Juniperus communis* var. | *n* | 30 | 150 | 100 | 100 |
| *saxatilis* | *0* | 18.8±5.7 | 1.9±0.4 | 37.0±44.5 | 19.4±23.8 |
|  | *1* | 16.0±1.9 | 1.8±0.4 | 8.8±1.6 | 5.1±1.5 |
|  | *p* | *** | n.s. | n.s. | *** |
| *Rhododendron ferrugineum* | *n* | 6 | 18 | 18 | 18 |
|  | *0* | 23.6±10.5 | 29.8±10.6 | 1061±762 | 35.2±31.8 |
|  | *1* | 7.1±0.4 | 16.9±2.8 | 134.4±18.5 | 8.0±0.5 |
|  | *p* | *** | *** | *** | ** |
| *Salix herbacea* | *n* | 6 | 18 | 18 | 18 |
|  | *0* | 2.5±0.7 | 6.5±3.1 | 108.4±35.3 | 17.9±3.1 |
|  | *1* | 1.9±0.4 | 5.9±2.3 | 101.0±35.6 | 17.4±2.7 |
|  | *p* | * | n.s. | n.s. | n.s. |
| *Salix serpilifolia* | *n* | 10 | 50 | 50 | 50 |
|  | *0* | 3.6±1.1 | 1.1±0.3 | 12.1±4.8 | 11.2±2.0 |
|  | *1* | 5.8±0.6 | 2.6±0.2 | 26.8±6.2 | 10.5±2.4 |
|  |  |  |  |  |  |
| *Thymus praecox* ssp. *polytrichus* | *n* | 10 | 50 | 50 | 50 |
|  | *0* | 15.6±6.6 | 1.1±0.1 | 15.4±2.4 | 13.8±1.7 |
|  | *1* | 7.9±3.6 | 1.1±0.8 | 70.3±71.7 | 58.3±60.0 |
|  | *p* | *** | n.s. | *** | *** |
| *Vaccinium vitis-idaea* | *n* | 20 | 97 | 50 | 50 |
|  | *0* | 20.8±2.5 | 22.2±7.6 | 147.4±53.3 | 6.7±1.3 |
|  | *1* | 14.7±4.4 | 25.2±10.8 | 151.2±57.0 | 6.3±1.1 |
|  | *p* | *** | n.s. | n.s. | n.s. |

|  | p<0.10 |  | p<0.05 |  | p<0.01 |  | p<0.001 |
| --- | --- | --- | --- | --- | --- | --- | --- |
